# Social inequalities in self-reported SARS-CoV-2 infection in Brazilian adults: PNAD COVID-19

**DOI:** 10.1590/1980-549720240042

**Published:** 2024-08-30

**Authors:** Mateus Andrade Rocha, Cândido Norberto Bronzoni de Mattos, Marcos Pascoal Pattussi

**Affiliations:** I Universidade do Vale do Rio dos Sinos, Postgraduate Program in Collective Health – São Leopoldo (RS), Brazil.; II Universidade Federal de Santa Catarina, Postgraduate Program in Dentistry – Florianópolis (SC), Brazil.

**Keywords:** Public health, COVID-19, Social inequity, Adults

## Abstract

**Objective:**

To investigate inequalities related to race/ethnicity and socioeconomic status in self-reported positive diagnosis for COVID-19 in Brazilian adults.

**Methods:**

Data available from the National Household Sample Survey COVID-19 (PNAD COVID 19) (July/September/November, 2020) were used in this retrospective investigation. The analyses considered the sampling design, primary sampling units, strata and sample weights. Poisson regression with robust variance was used to estimate prevalence ratio (PR) and the 95% confidence interval (95%CI) of the associations.

**Results:**

In July, September and November 2020, with regard to the rapid test, indigenous people were 2.45 (95%CI 1.48–4.08), 2.53 (95%CI 1.74–4.41) and 1.23 (95%CI 1.11–1.86) times more likely to report a positive history of SARS-CoV-2 infection, respectively. With regard to the RT-PCR test in November, indigenous people were more likely to test positive for COVID-19 (PR: 1.90; 95%CI 1.07–3.38). It was observed that the indigenous group was 1.86 (95%CI 1.05–3.29) and 2.11 (95%CI 1.12–3.59) times more likely to test positive for COVID-19 in September and November (2020). Income was associated with testing positive for COVID-19: in November, individuals whose income ranged from R$0.00–R$1.044 were more likely (PR: 1.69; 95%CI 1.16–23.06) to test positive using the RT-PCR test; participants whose income was in this range were also more likely to be diagnosed with COVID-19 using blood tests (PR: 1.72; 95%CI 1.43–2.07).

**Conclusion:**

The data presented show an association between race/ethnicity and economic status with a positive diagnosis of COVID-19.

## INTRODUCTION

In the first trimester of 2020, a new highly transmissible and pathogenic type of COVID-19 coronavirus was responsible for infecting a large number of individuals globally, triggering the COVID-19 pandemic^1^. The COVID-19 virus affects the respiratory system, causing mild symptoms in many people, but it can lead to critical conditions in a percentage of cases, with massive alveolar damage and respiratory failure, which can contribute with death^2,3^. Brazil emerged as the pandemic epicenter of the coronavirus disease. Only during the first wave of the pandemic (from March to November, 2020), more than 7.9 million cases and more than 100 thousand deaths caused by the disease were registered in the country^4^. The infection diagnosis can be carried out through a variety of tests, with oral and blood fluids. However, the gold-standard diagnostic method for COVID-19 is based on a reverse transcription polymerase chain reaction (RT-PCR) molecular test, aiming at detecting viral RNA in respiratory samples, such as nasopharyngeal swabs or bronchial aspirate^5^.

Evidence shows that being male, at a more advanced age, with unhealthy habits (for example, smoking), obesity and diagnosis of chronic diseases (for example, hypertension, diabetes and respiratory and cardiovascular diseases) present higher risk of infection and evolution to a critical or mortal disease status^6–9^. Besides the factors related with individual organic conditions, the risks of infection and its severe course are distributed unequally in society^4^. International literature, especially in developed countries, reports higher impact of the disease in people at low socioeconomic levels, and in minority racial/ethnic groups^10,11^.

Recently, a population-base Brazilian study observed that indigenous populations, large families and families with low socioeconomic status had higher prevalence of antibodies for SARS-CoV-2 in comparison to the white population, small families and with high socioeconomic status^12^. Cross-sectional findings also showed that lower schooling and income and higher number of individuals in the household were strongly associated with higher mortality rates caused by COVID-19^13^. In this sense, the objective of this study was to investigate race and monthly income inequalities in the self-reported infection by SARS-CoV-2 in adults during the first wave of the pandemic in Brazil.

## METHODS

This repeated cross-sectional study was performed with the data made available by the National Household Sample Survey COVID-19 (PNAD). The objective was to estimate the number of people with symptoms related to flu-like syndrome and to monitor the impacts of the COVID-19 pandemic in the Brazilian labor market^14^. This study's report was performed according to the orientations from the Strengthening the Reporting of Observational Studies in Epidemiology (Strobe) Statement^15^. The use of public secondary data in this study exempts the need for approval of the use of this information by the Research Ethics Committee.

The data were collected by approximately two thousand agents from the Brazilian Institute of Geography and Statistics (IBGE), based on structured interviews via telephone in about 193.6 thousand households, distributed in 3,364 cities in all Brazilian macroregions. Data collection took place between May and November, 2020, during the COVID-19 pandemic in Brazil^12^. For this study, we used data from July, September and November, 2020.

The selection and training of the research team were performed by the Coordination of Training and Improvement of the National School of Sciences and Statistics from IBGE. The training was composed of two content modules; one regarding the approach to the participant in the telephone call, and the other about the application of the research survey. The complete methodological process of PNAD COVID-19 can be accessed in previous studies^14,16,17^.

Participants were invited to answer the following questions:

1. "*Have you been diagnosed with the COVID-19 virus*" (yes; no; I cannot answer that);
2. *"Have you taken any test to know if you were infected with coronavirus"* (yes; no; I cannot answer that);
3. "*What test was performed to verify if you had COVID-19?"* (swab collection from the mouth/nose RT-PCR; through fingerstick (fast test); blood collection through the vein in the arm (blood test).

For each test, there was a single question regarding the history of testing for COVID-19 (yes or no). Inconclusive responses, without results or results that were ignored in the tests, were excluded. Positive answers in the utilized tests were the study outcomes.

Characteristics related to race/ethnicity and family income of the individuals were also obtained. The monthly family income of participants was collected in absolute numbers (Brazilian Real – R$), and then classified according to the distribution per quartiles, being: R$≥R$ 2,500; R$ 2,499–R$ 1,430; R$ 1,429–R$ 1,045; R$1,044–0. Race/ethnicity of participants was identified according to the criteria of IBGE and considered white, black, yellow, mixed and indigenous participants. Other exposure variables explored sociodemographic aspects related to social distancing and owning cleaning and protection items, which were also considered as confounding factors in this study: age in complete years (categorized in age groups based on criteria from: 18–29; 30–39; 40–49; 50–59; ≥60 years of age), gender (male; female), schooling (complete/incomplete higher education; complete high school; complete/incomplete elementary school; incomplete elementary school/no schooling), morbidities (no morbidities; 1 morbidity; 2 morbidities; ≥3 morbidities), utensils alcohol (does not have alcohol; has alcohol), utensils mask (does not have masks; has masks), distancing (strictly isolated; going out only for basic needs; going out for work or essential activities; did not practice distancing). The study's questionnaire containing the variables used in this study is found in Appendix 1.

All statistical analyses were conducted using the Stata Statistical Package (Version 16.0) (Stata Corp, College Station, Texas, USA). All data supporting the findings in this study are available with the corresponding author with a previous request, according to the FAIR Data Principles (www.force11.org/group/fairgroup/fairprinciples). The variables were described through relative and absolute frequencies. Poisson Regression with robust variance was used to estimate prevalence ratio (PR) and 95% confidence interval (95%CI) in the association between outcomes and exposure variables. In the multivariate analysis, exposure variables were controlled by confounding factors associated with the outcome at a significance level lower than 10%. The analyses considered sampling design, primary sampling units, strata and sampling weights. A significance level lower than 5% was chosen to consider associations between exposure and outcome as being significant.

## RESULTS

Generally, the performance of any test for COVID-19 was reported by 26.673 (July); 35,587 (September); and 45,180 (November) participants. Taking a RT-PCR test for COVID- 19 in July, September and November was reported 7,026, 12,943 and 18,308 participants, respectively. In the same period, 49,407 rapid tests were performed, as follows: July: n=11,630; September: n=16,954; November: n=20,823. Also, the history of blood tests was reported by 6,886 (July), 10,668 (September) and 13,102 (November) interviewed individuals. Tables 1 e 2 present the sampling distribution of exposure variables according to the history of infection by SARS-CoV-2 per testing modalities.

**Table 1:** Sample description according to demographic and socioeconomic variables related to the prevalence of positive RT-PCR results in July (n=7,026), September (n=12,943) and November (n=18,308), and COVID-19 rapid test in July (n=11,630), September (n=16,954) and November (n=20,823), 2020.

Variables		July		September		November	
		n (%)	+COVID (95%CI)*	n (%)	+COVID (95%CI)*	n (%)	+COVID (95%CI)*
RT-PCR							
	Total	7,026 (100)	27.8 (26.0–29.6)	12,943 (100)	28.3 (27.8–28.7)	18,308 (100)	29.3 (28.9–29.7)
Race							
	White	3,320 (47.3)	26.6 (24.3–29.0)	6,245 (48.3)	26.9 (26.4–27.3)	9,201 (50.3)	28.1 (26.2–30.0)
	Black	601 (8.6)	27.8 (23.1–33.0)	1,139 (8.8)	27.5 (27.0–28.1)	1,570 (8.6)	27.3 (23.7–30.8)
	Mixed race	3,028 (43.1)	29.4 (26.9–32.1)	5,420 (41.9)	30.7 (28.6–32.8)	7,361 (40.2)	31.7 (29.9–33.4)
	Yellow	52 (0.7)	22.7 (7.8–50.4)	98 (0.8)	14.4 (9.6–199.2)	120 (0.7)	20.0 (15.4–24.6)
	Indigenous	22 (0.3)	35.8 (13.8–66.0)	38 (0.3)	39.5 (31.6–47.4)	52 (0.3)	40.0 (34.5–46.3)
Age group (years)							
	18–29	1,317 (20.3)	24.4 (21.4–27.8)	2,722 (21.0)	26.3 (22.7–29.8)	3,923 (21.4)	27.1 (25.2–28.9)
	30–39	1,711 (26.3)	29.1 (26.0–32.3)	3,126 (24.2)	29.5 (26.9–32.1)	4,355 (23.8)	31.2 (29.4–32.9)
	40–49	1,443 (22.2)	29.2 (26.1–32.6)	2,847 (22.0)	29.4 (26.3–325)	3,953 (21.6)	30.8 (28.5–33.1)
	50–59	1,065 (16.4)	32.4 (28.5–36.5)	2,173 (16.8)	29.3 (25.4–33.2)	3,018 (16.5)	31.2 (28.9–33.5)
	≥60	959 (14.8)	26.1 (22.2–30.4)	2,075 (16.0)	27.6 (23.6–31.6)	3,059 (16.7)	26.5 (24.4–28.6)
Gender							
	Male	3,240 (46.1)	27.7 (25.6–30.0)	5,852 (45.2)	28.7 (25.3–31.3)	8,260 (45.1)	29.7 (27.1–32.4)
	Female	3,786 (53.9)	27.8 (25.7–29.9)	7,091 (54.8)	28.3 (26.8–29.7)	10,048 (54.9)	29.3 (28,1–30,5)
Schooling							
	Higher Education	2,885 (41.1)	25.9 (23.7–28.3)	5,419 (41.9)	27.0 (23.8–30.2)	7,899 (43.1)	29.1 (27.1–31.0)
	Complete HS	1,957 (27.8)	31.6 (28.7–34.6)	3,891 (30.1)	30.6 (26.5–34.7)	5,427 (29.6)	30.9 (28.2–33.6)
	Complete ES/incomplete ES	822 (11.7)	29.9 (25.5–34.6)	1,567 (12.1)	28.9 (23.6–34.1)	2,166 (11.8)	29.8 (27.1–32.5)
	Incomplete ES/No schooling	1,362 (19.4)	25.1 (21.6–28.9)	2,066 (16.0)	27.9 (23.6–32.1)	2,816 (15.4)	27.6 (24.7–30.5)
Income							
	≥R$ 2,500	1,993 (43.7)	28.1 (25.3–31.1)	3,610 (41.0)	27.3 (24.3–30.3)	5,055 (41.0)	30.0 (27.8–32.1)
	R$ 2,499–R$ 1,430	1,183 (25.9)	27.6 (24.4–31.1)	2,292 (26.1)	28.9 (25.6–32.1)	3,189 (25.9)	29.4 (26.2–32.6)
	R$ 1,429–R$ 1,045	1,081 (23.7)	27.6 (24.2–31.3)	2,212 (25.1)	29.6 (26.1–33.1)	3,103 (25.2)	30.6 (28.2–33.1)
	R$ 1,044–0	307 (6.7)	25.3 (18.7–33.3)	682 (7.8)	26.7 (20.3–33.1)	968 (7.9)	27.7 (21.9–33.4)
Rapid test							
	Total	11,630 (100)	16.2 (15.1–17.3)	16,954 (100)	19.7 (18.5–20.8)	20,823 (100)	19.9 (18.7–21.8)
Race							
	White	4,707 (40.5)	13.1 (11.8–14.6)	7,033 (41.5)	16.4 (13.9–18.8)	8,771 (42.1)	17.0 (14.9–19.0)
	Black	1,035 (8.9)	16.3 (13.1–20.1)	1,622 (9.6)	20.4 (17.3–23.5)	1,962 (9.4)	19.3 (16.7–21.9)
	Mixed race	5,748 (49.4)	18.9 (17.3–20.6)	8,108 (47.8)	22.3 (20.6–24.1)	9,856 (47.3)	22.2 (20.9–23.5)
	Yellow	75 (0.7)	12.7 (5.7–26.1)	86 (0.5)	18.6 (4.6–32.6)	117 (0.6)	18.8 (3.8–33.8)
	Indigenous	61 (0.5)	34.9 (20.9–52.2)	101 (0.6)	36.6 (22.8–50.4)	113 (0.5)	22.1 (11.1–33.1)
Age group (years)							
	18–29	2,085 (19.5)	15.2 (13.2–17.4)	3,418 (20.2)	19.7 (17.2–22.2)	4,127 (19.8)	19.8 (17.9–21.7)
	30–39	2,676 (25.0)	15.9 (14.0–17.9)	3,977 (23.5)	19.2 (17.0–21.4)	4,716 (22.6)	20.2 (18.6–21.8)
	40–49	2,397 (22.4)	17.0 (15.1-19.1)	3,716 (21.9)	21.6 (19.7–23.5)	4,581 (22.0)	21.6 (19.9–23.3)
	50–59	1,871 (17.5)	16.0 (14.0–18.2)	2,924 (17.2)	19.6 (16.0–23.2)	3,676 (17.7)	18.6 (16.4–20.8)
	≥60	1,659 (15.5)	17.4 (14.9–20.2)	2,919 (17.2)	18.3 (14.7–21.8)	3,723 (17.9)	17.8 (15.2–20.4)
Gender							
	Male	5,470 (47.0)	15.2 (13.9–16.6)	7,819 (46.1)	18.6 (16.9–20.3)	9,604 (46.1)	18.9 (17.4–20.5)
	Female	6,160 (53.0)	17.0 (15.7–18.4)	9,135 (53.9)	20.7 (19.0–22.4)	11,219 (53.9)	20.4 (18.8–-21.9)
Schooling							
	Higher Education	3,817 (32.8)	12.7 (11.3–14.2)	5,729 (33.8)	17.2 (16.3–18.1)	6,961 (33.4)	17.7 (16.8–18.6)
	Complete HS	3,391 (29.2)	18.2 (16.4–20.1)	5,316 (31.4)	20.7 (19.6–21.7)	6,422 (30.8)	20.9 (19.9–21.9)
	Complete ES/incomplete ES	1,578 (13.6)	19.3 (16.9–22.0)	2,107 (12.4)	22.1 (20.4–23.7)	2,667 (12.8)	21.8 (20.3–23.3)
	Incomplete ES/No schooling	2,844 (24.4)	17.0 (15.1–19.1)	3,802 (22.4)	20.9 (19.6–22.2)	4,773 (22.9)	19.8 (18.7–20.9)
Income							
	≥R$ 2,500	2,563 (36.3)	12.3 (10.6–14.2)	3,682 (34.4)	15.7 (13.8–17.5)	4,431 (34.0)	16.5 (14.4–18.6)
	R$ 2,499 – R$ 1,430	1,828 (25.8)	15.4 (13.3–17.8)	2,727 (25.5)	18.9 (17.3–20.5)	3,253 (25.0)	19.3 (17.5–21.1)
	R$ 1,429- R$ 1,045	1,993 (28.2)	16.8 (14.8–19.1)	3,076 (28.7)	20.6 (18.9–22.2)	3,764 (28.9)	20.5 (18.5–22.6)
	R$ 1,044 – 0	687 (9.7)	19.2 (15.6–23.5)	1,224 (11.4)	19.9 (16.7–23.1)	1,580 (12.1)	19.5 (15.5–23.5)

* Prevalence rates and 95% confidence intervals for positive RT-PCR tests or rapid tests for COVID-19.

CI: confidence interval; HS: high school; ES: elementary school.

**Table 2:** Sample description according to demographic and socioeconomic variables related to the prevalence of positive blood test for COVID-19 in July (n=6,886), September (n=10,668) and November (n=13,102), 2020.

Variables		July		September		November	
		n (%)	+COVID (95%CI)*	n (%)	+COVID (95%CI)*	n (%)	+COVID (95%CI)*
Total		6,886 (100)	25.8 (24.2–27.5)	10,668 (100)	28.5 (27.3–29.7)	13,102 (100)	28.2 (27.4–28.9)
Race							
	White	3,040 (44.2)	21.7 (19.6–23.9)	4,637 (43.5)	24.2 (22.3–26.1)	5,828 (44.5)	25.1 (23.4–26.8)
	Black	530 (7.7)	23.6 (19.2–28.6)	876 (8.2)	29.3 (27.1–31.5)	1,068 (8.2)	28.3 (26.4–30.2)
	Mixed race	3,217 (46.7)	31.3 (28.8–33.9)	5,026 (47.1)	33.0 (21.6–34.4)	6,040 (46.1)	31.8 (20.5–33.1)
	Yellow	54 (0.8)	22.2 (9.5–43.6)	75 (0.7)	12.0 (3.1–20.9)	96 (0.7)	15.8 (6.9–24.7)
	Indigenous	44 (0.6)	30.4 (14.2–53.6)	52 (0.5)	37.3 (22.3–52.3)	65 (0.5)	42.2 (27.2–57.2)
Age group (years)							
	18–29	1,138 (17.8)	24.1 (21.0–27.5)	1,971 (18.5)	27.6 (25.5–29.7)	2,387 (18.2)	28.1 (26.2–30.0)
	30–39	1,581 (24.8)	26.3 (23.2–29.6)	2,465 (23.1)	28.4 (26.4–30.4)	2,911 (22.2)	29.6 (27.8–31.4)
	40–49	1,441 (22.6)	26.7 (23.6–30.0)	2,263 (21.2)	29.2 (26.9–31.5)	2,845 (21.7)	28.3 (26.2–30.4)
	50–59	1,121 (17.5)	24.5 (21.3–28.0)	1,934 (18.1)	29.7 (26.9–32.5)	2,384 (18.2)	28.5 (26.7–30.3)
	≥60	1,103 (17.3)	27.0 (23.5–30.8)	2,035 (19.1)	28.9 (26.5–31.3)	2,575 (19.7)	27.4 (25.8–29.0)
Gender							
	Male	3,185 (46.2)	24.8 (22.7–27.0)	4,892 (45.9)	28.2 (26.3–30.1)	5,954 (45.4)	27.6 (25.9–30.6)
	Female	3,701 (53.8)	26.7 (24.8–28.7)	5,776 (54.1)	29.2 (27.7–30.7)	7,148 (54.6)	29.1 (27.5–30.7)
Schooling							
	Higher Education	2,733 (39.7)	20.6 (18.6–22.9)	4,307 (40.4)	24.4 (22.6–26.2)	5,317 (40.6)	25.1 (23.6–26.6)
	Complete ES	1,812 (26.3)	30.6 (27.7–33.7)	3,042 (28.5)	30.7 (28.4–33.0)	3,667 (28.0)	30.0 (28.1–31.9)
	Complete ES/incomplete ES	815 (11.8)	28.1 (24.0–32.5)	1,177 (11.0)	33.4 (30.7–36.1)	1,491 (11.4)	31.4 (29.0–33.6)
	Incomplete ES/no schooling	1,526 (22.2)	29.6 (26.3–-33.1)	2,142 (20.1)	32.1 (29.5–34.7)	2,627 (20.1)	31.3 (29.5–33.3)
Income							
	≥R$ 2,500	1,888 (43.9)	19.1 (16.7–21.8)	2,869 (41.3)	22.9 (20.8–25.0)	3,420 (40.5)	23.4 (21.5–25.3)
	R$ 2,499–R$ 1,430	964 (22.4)	25.6 (22.1–-29.5)	1,619 (23.3)	28.7 (25.5–31.9)	1,994 (23.6)	27.9 (24.9–30.9)
	R$ 1,429–R$ 1,045	1,088 (25.3)	25.0 (21.9–28.4)	1,772 (25.5)	28.8 (25.9–31.7)	2,162 (25.6)	28.6 (26.4–30.8)
	R$ 1,044–0	364 (8.4)	31.1 (25.0–37.8)	684 (9.9)	33.0 (27.3–38.7)	872 (10.3)	29.7 (25.5–33.9)

* Prevalence rates and 95% confidence intervals for positive RT-PCR tests or rapid tests for COVID-19.

CI: confidence interval; HS: high school; ES: elementary school.

Table 3 presents PR and 95% CI of the crude and adjusted analyses between the performance of the three different tests (RT-PCR, rapid test and blood test) during the months of July, September and November (2020) and the exposure variables. After adjusting for potential confounding factors, a significant effect of race/ethnicity on COVID-19 testing via RT-PCR was observed among Indigenous participants in November (PR: 1.90); 95%CI 1.07–3.38), considering that this group presented higher chances of infection by SARS-CoV-2 via RT-PCR in comparison do white individuals. Participants of mixed race/ethnicity showed a higher PR of positive RT-PCR tests in September (PR: 1.20; 95%CI 1.10–1.30), in comparison to white people. Also, individuals with lower income R$ 0–1) had more chances of presenting a RT-PCR test to verify the infection by COVID-19 than those with higher family income (≥R$ 2,500) in September (PR: 1.87; 95%CI 1.15–2.67) and November (PR: 1.69; 95%CI 1.16–3.06).

**Table 3:** Crude and adjusted analyses for positive RT-PCR, rapid test and blood test for COVID-19 in July, September and November, 2020.

Variables		July		September		November	
		_CRUDE_PR (95%CI)	_ADJUSTED_PR (95%CI)	_CRUDE_PR (95%CI)	_ADJUSTED_PR (95%CI)	_CRUDE_PR (95%CI)	_ADJUSTED_PR (95%CI)
RT-PCR Test							
Race							
	White	1.00	1.00	1.00	1.00	1.00	1.00
	Black	1.05 (0.86–1.27)	1.09 (0.88–1.35)	1.06 (0.92–1.22)	1.05 (0.91–1.21)	0.96 (0.85–1.09)	0.97 (0.86–1.10)
	Mixed race	1.11 (0.98–1.25)	1.05 (0.92–1.21)	1.21 (1.11–1.31)	1.20 (1.10–1.30)	1.18 (1.11–1.27)	1.20 (1.12–1.29)
	Yellow	0.85 (0.34–2.16)	0.72 (0.26–1.99)	0.46 (0.26–0.81)	0.44 (0.25–0.77)	0.65 (0.41–1.02)	0.65 (0.41–1.02)
	Indigenous	1.35 (0.65–2.79)	1.39 (0.37–5.14)	1.73 (1.09–3.33)	1.63 (0.85–3.15)	1.92 (1.08–3.40)	1.90 (1.07–3.38)
Income							
	≥R$ 2,500	1.00	1.00	1.00	1.00*	1.00	1.00
	R$ 2,499–R$ 1,430	0.98 (0.84–1.15)	0.90 (0.76–1.05)	1.09 (0.97– 1.23)	1.05 (0.93–1.19)	0.98 (0.89–1.08)	0.97 (0.87–1.08)
	R$ 1,429–R$ 1,045	0.98 (0.83–1.16)	0.90 (0.75–1.07)	1.14 (1.01–1.28)	1.08 (0.94–1.24)	1.05 (0.95–1.15)	1.04 (0.93–1.16)
	R$ 1,044–0	0.90 (0.66–1.22)	0.78 (0.56–1.08)	0.99 (0.82–1.19)	1.87 (1.15–2.67)	1.31 (0.98–1.76)	1.69 (1.16–3.06)
Rapid test							
Race							
	White	1.00	1.00/	1.00*	1.00	1.00	1.00*
	Black	1.24 (0.98–1.57)	1.09 (0.84–1.42)	1.31 (1.15–1.51)	1.31 (1.02–1.32)	1.17 (1.04–1.33)	1.16 (1.03–1.32)
	Mixed race	1.44 (1.26–1.64)	1.22 (1.04–1.44)	1.47 (1.35–1.59)	1.47 (1.35–1.60)	1.42 (0.91–2.26)	1.41 (0.90–2.21)
	Yellow	0.97 (0.45–2.07)	1.04 (0.43–2.51)	1.17 (0.67–2.02)	1.07 (0.58–1.87)	1.16 (0.73–1.85)	1.15 (0.72–1.84)
	Indigenous	2.66 (1.69–4.19)	2.45 (1.48–4.08)	2.95 (1.96–4.45)	2.53 (1.74–4.41)	1.39 (1.29–1.50)	1.23 (1.11–1.86)
Income							
	≥R$ 2,500	1.00	1.00	1.00*	1.00*	1.00	1.00*
	R$ 2,499–R$ 1,430	1.25 (1.02–1.53)	1.14 (0.93–1.41)	1.26 (1.11–1.44)	1.27 (1.11–1.45)	1.19 (1.07–1.36)	1.22 (1.08–1.37)
	R$ 1,429–R$ 1,045	1.36 (1.13–1.65)	1.16 (0.94–1.43)	1.39 (1.22–1.58)	1.42 (1.25–1.61)	1.31 (1.17–1.47)	1.33 (1.19–1.49)
	R$ 1,044–0	1.56 (1.22–2.00)	1.20 (1.00–1.57)	1.35 (1.13–1.59)	1.38 (1.17–1.64)	1.22 (1.06–1.42)	1.25 (1.08–1.45)
Blood test							
Race							
	White	1.00	1.00	1.00	1.00	1.00	1.00*
	Black	1.09 (0.87–1.36)	1.07 (0.82–1.38)	1.30 (1.11–1.53)	1.30 (1.11–1.53)	1.18 (1.02–1.37)	1.18 (1.02–1.37)
	Mixed race	1.44 (1.28–1.63)	1.30 (1.11–1.53)	1.84 (1.41–1.69)	1.54 (1.41–1.69)	1.39 (1.28–1.51)	1.39 (1.28–1.51)
	Yellow	1.02 (0.48–2.18)	0.78 (0.31–1.99)	0.43 (0.21–0.86)	0.43 (0.21–0.86)	0.56 (0.32–0.98)	0.56 (0.32–0.98)
	Indigenous	1.40 (0.74–2.68)	1.14 (0.53–2.48)	1.36 (1.02–3.29)	1.86 (1.05–3.29)	2.18 (1.32–3.59)	2.11 (1.12–3.59)
Income							
	≥R$ 2,500	1.00	1.00	1.00	1.00*	1.00	1.00*
	R$ 2,499–R$ 1,430	1.34 (1.10–1.64)	1.28 (1.05–1.56)	1.36 (1.18–1.56)	1.38 (1.20–1.59)	1.27 (1.12–1.44)	1.28 (1.13–1.45)
	R$ 1,429–R$ 1,045	1.31 (1.09–1.57)	1.21 (1.00–1.46)	1.37 (1.19–1.57)	1.41 (1.22–1.62)	1.31 (1.16–1.48)	1.34 (1.18–1.52)
	R$ 1,044–0	1.63 (1.27–2.08)	1.39 (1.08–1.79)	1.67 (1.39–2.00)	1.72 (1.43–2.07)	1.38 (1.17–1.63)	1.43 (1.21–1.69)

* Adjusted for schooling, gender and age group

Self-identified Indigenous participants had a higher likelihood of being diagnosed with COVID-19 through rapid tests compared to white participants in all months of follow-up: July (RP: 2.45; 95%CI 1.48–4.08), September (PR: 2.53; 95%CI 1.74–4.41), November (PR: 1.23; 95%CI 1.11–1.86). Monthly average income of participants was associated with the prevalence of COVID-19 rapid tests: participants with lower monthly income presented higher chances of undergoing a fast test than those who earn ≥2,500 reais per month in July, September and November (2020). It was identified that mixed race participants had higher prevalence of positive testing for COVID-19 through blood tests in all months of follow-up: July (PR: 1,30; 95%CI 1.11–1.53), September (PR: 1.54; 95%CI 1.41–1.69), November (PR: 1.39; 95%CI 1.28–1.51), compared to white individuals. Individuals self-identified as Indigenous also showed a higher prevalence ratio of positive COVID-19 testing through blood tests, but only in September (PR: 1.86; 95%CI 1.05–3.29) and November (PR: 2.11; 95%CI 1.12–3.59), 2020. The lower monthly family income was associated with the lower chances of positive results in the blood test in all months of follow-up than those whose income was ≥R$ 2,500/month (Table 3).

## DISCUSSION

This study used data from PNAD COVID-19 to investigate the inequalities related to race and income in the history of COVID-19 testing according to diagnostic modalities in adults during the first wave of the pandemic in Brazil. The results indicate that Indigenous individuals and those with lower average monthly income were more likely to be diagnosed with COVID-19, regardless of the test used and the examination period.

In this study, non-white individuals, especially those of mixed race and/or Indigenous descent, exhibited higher seroprevalence for COVID-19 compared to white subjects.

This finding can be attributed to the social conditions to which minority populations are exposed, predisposing them to higher rates of infection with the disease in this group ^18^. This finding corroborates the historical vulnerability experienced by the Indigenous population, in particular, which has faced higher rates of infection in the past, such as during the Spanish flu, H1N1 virus infection, and SARS-CoV^19^.

Indeed, this population group is heavily impacted by inequities in social determinants of health. In addition to cultural and geographical barriers, Indigenous people experience higher levels of poverty, malnutrition, lower schooling, difficulty accessing health services, and precarious basic sanitation systems^19,20^. Furthermore, it is important to highlight the common characteristic of geographic isolation in this population as a potential barrier to accessing healthcare measures aimed at combating the pandemic. Furthermore, immunological factors specific to Indigenous populations and the presence of chronic diseases increase susceptibility to outbreaks of infectious diseases, rendering these peoples more vulnerable ^21^. This reality is exacerbated when the exposure of this community is linked to governmental neglect in addressing the disease, as reported by the National Health Council (CNS) and the National Human Rights Council (CNDH)^22^.

The results of this study highlight a significant association between testing via RT-PCR and blood tests and the monthly income of participants. Individuals with lower income were more likely to test positive using these diagnostic modalities. In Brazil, the spread of the COVID-19 virus began among individuals from higher economic classes, and subsequently, the virus spread rapidly among people from less privileged economic backgrounds, as some of these individuals continued their daily activities out of necessity for subsistence^23,24^. Studies have reported that unfavorable socioeconomic conditions, lower levels of education, and a higher number of household residents may predispose individuals to a higher rate of COVID-19 infection^12^. The Economic Commission for Latin America and the Caribbean (ECLAC) suggests that the impoverishment of the Brazilian population in recent years may have increased the impact of COVID-19 in the country ^25^. Disadvantaged groups tend to have less structured occupations and insufficient income for survival^26–28^, often engaged in jobs that do not allow remote work, requiring the use of public transportation for commuting and thus increasing contact among people^29,30^. Furthermore, insufficient testing conducted in Brazil during the period of the PNAD COVID-19, due to the limited number of tests available in the public health system (SUS) and the Brazilian government's failure to procure them, resulted in individual purchases of tests, thereby excluding disadvantaged populations from disease diagnosis^26,28,29^. However, in the outcomes of the tests conducted, higher positivity rates are highlighted in the economically more vulnerable population.

The results presented in this study should be interpreted in light of its limitations. The main limitation of this investigation refers to its cross-sectional nature, which does not allow for causal inference in the identified associations. The use of self-reported data can also be considered a limitation, as subjective perception is influenced by verbal behavior, which in turn is reinforced by the individual's environment^25^.

Similarly, it is important to highlight limitations related to test results. The RT-PCR test is recommended for symptomatic patients in the acute phase of the disease between the third and seventh day. Therefore, potential false negatives should be considered, which can occur when the amount of collected viral genome is insufficient or when the viral replication window period is missed^5^. However, this test has high sensitivity and specificity: 97.2% and 98.9%, respectively^5^.

Furthermore, COVID-19 diagnosis can be achieved based on the immune response to SARS-CoV-2 infection, using immunochromatographic serological tests for rapid detection of IgG/IgM antibodies in blood, serum, or plasma samples from individuals^5^. The main limitation of this tool is the requirement for testing from the eighth day after the onset of symptoms. Therefore, disease detection based on this detection method may occur during a period of patient recovery, posing a challenge for surveillance and transmission control services. The IgM and IgG serological tests have a sensitivity of 84.5% and specificity of 91.6%^4,5^. However, immunochromatographic rapid tests showed low sensitivity, particularly in the early stages of the pandemic^31^.

It is important to note that the sensitivity of a diagnostic test refers to its ability to correctly identify positive cases of the disease, while specificity indicates its ability to correctly identify negative results^4,5^.

Based on a literature review conducted by the authors, there have been no studies published so far that have sought to validate these self-reported positive COVID-19 results. Cohort studies with biochemical tests could better illustrate the disparities in disease transmission. Besides, it's important to consider that in April 2020, the most vulnerable indigenous lands were those located on the outskirts of major urban centers such as Manaus, the Rio Branco-Porto Velho axis, Fortaleza, Salvador, and capitals in the South and Southeast of Brazil. Therefore, considering the scope of the PNAD COVID-19 in major urban centers, these findings may represent a partial picture of reality, as they do not effectively reach villages and indigenous lands in isolated regions of the country. Finally, the results described here are valid and robust, contributing to a field that has been relatively underexplored in Brazilian literature.

This study identified a significant association between ethnicity/race and economic status with positive COVID-19 outcomes among Brazilian adults. This situation reflects vulnerability in these groups and underscores the need for the development and expansion of more equitable public policies that address the needs of vulnerable groups during a public health crisis.

## Supplementary Material


