# Supplementary material for: Social inequalities in self-reported SARS-CoV-2 infection in Brazilian adults: PNAD COVID-19
Source: Rev Bras Epidemiol. 2024 Aug 30;27:e240042. doi: 10.1590/1980-549720240042 (PMC11383518; doi:10.1590/1980-549720240042)
Supplement: Supplementary file 1 [file 1980-5497-rbepid-27-e240042-Suppl01.pdf]

PNAD COVID19 – Questionário de julho, agosto, setembro e outubro

Blocos:

**Modulo A -** Características dos moradores - *Vamos trazer da PNAD Contínua*	**11 Perguntas**
**Módulo B -** COVID-19	**11 Perguntas**
**Módulo C -** Características de trabalho das pessoas de 14 anos ou mais de idade	**24 Perguntas**
**Módulo D -** Rendimentos de outras fontes de todos os moradores do domicílio	**1 Pergunta**
**Módulo E -** Empréstimos	**2 Perguntas**
**Módulo F** - Domicílio, propriedade e valor do aluguel	**4 Perguntas**

Público alvo: Todos os moradores residentes nos domicílios selecionados para responder a pesquisa

Forma de coleta da informação: Entrevista exclusivamente por telefone

Período de coleta: De maio a setembro de 2020

Objetivos da PNAD COVID:

Módulo A: Listar os moradores do domicílio e suas características pessoais (idade, sexo, cor e escolaridade).

Módulo B: Levantar informações sobre:
- Identificação de indivíduos com sintomas de síndrome gripal e COVID-19;
- Busca por estabelecimento de saúde pública;
- Tipo providência tomada para ser recuperar desses sintomas;
- Identificar o estabelecimento de saúde demandado em função dos sintomas listados;
- Identificar se houve internação na busca do estabelecimento de saúde;
- Procedimentos adotados durante o processo de internação;
- Posse de plano de saúde.

Módulo C: Levantar informações sobre:
- Contingente na força de trabalho (ocupados + desocupados);
- Contingente fora da força de trabalho;
- Algumas características de trabalho da população ocupada
- Força de trabalho ocupada afastada em função do distanciamento social;
- Contingente de ocupados em trabalho remoto;
- Contingente da população subutilizada;
- Contingente de desalentados;

- Contingente na informalidade;
- Ocupação e Atividade do trabalho
- Rendimento da população ocupada;
- Horas Trabalhadas
- Rendimento de Trabalho
- Contribuição para previdência
- Motivo deste afastamento temporário
- Local de exercício do trabalho
- Tamanho do empreendimento

Módulo D: Levantar informações referentes ao rendimento oriundo de outras fontes, de todos os moradores do domicílio.

Observação: a pesquisa tem ainda como objetivo atender demandas de alguns indicadores da Coordenação de Contas Nacionais para o cálculo do PIB Trimestral; da Coordenação de Índice de Preços para o cálculo da inflação; e da Coordenação de Trabalho e Rendimento para cálculo do RDPC para o FPE.

Módulo E: Identificar os domicílios em que ao menos um dos moradores precisou pedir empréstimo para arcar com as despesas durante a pandemia, sejam despesas familiares ou de suas empresas, para os empregadores e trabalhadores por conta própria.

Módulo F: Captar o valor de aluguel para o Sistema de Contas Nacionais e verificar a disponibilidade de alguns materiais de limpeza e proteção nos domicílios.

PNAD COVID19

Preenchimento automático:

Ano |__|__| mês |__|__| semana|__|

Unidade da Federação: |___|___| Município: |___|___|___|___|___|

Distrito: |___|___| Subdistrito: |___|___|

Endereço: ___________________________   CEP: |___|___|___|___|___|-|___|___|___|

Número do setor: |___|___|___|___|___|___|___|___|___|___|___|___|___|___|___|

Número de ordem do domicílio: |___|___|___|___|

SIAPE do Entrevistador: |___|___|___|___|___|___|___|___|

SIAPE do Supervisor: |___|___|___|___|___|___|___|___|

Telefone: |___|___| | ___|___|___|___|___|___|___|___|___|

Código	Ocorrência (tentativas)
	**Foi possível falar com algum morador elegível do domicílio, e iniciar a entrevista**
01	Iniciar a entrevista
	**Não foi possível estabelecer contato com um morador do domicílio**
02	Telefone não atende
03	Caixa postal / secretária eletrônica
04	Não foi possível completar a ligação
05	Telefone ocupado
06	Telefone fora de área / desligado
07	Sinal de fax
	**Foi possível falar com algum morador do domicílio, porém a entrevista não foi feita ou concluída**
08	Recusa do morador em participar da pesquisa
09	Telefone não corresponde a nenhum morador do domicílio ~~do endereço~~ selecionado
10	Respondente informa que telefone é de unidade não residencial
11	Respondente é criança (menor de 14 anos) e não há adulto presente
12	Agendada para outra data/hora
13	Outro motivo de interrupção

Data: __ / __ / _____ Horário: ____ : ____ Código da ocorrência: |__|__|

Data: __ / __ / _____ Horário: ____ : ____ Código da ocorrência: |__|__|

Data: __ / __ / _____ Horário: ____ : ____ Código da ocorrência: |__|__|

Data: __ / __ / _____ Horário: ____ : ____ Código da ocorrência: |__|__|

Data: __ / __ / _____ Horário: ____ : ____ Código da ocorrência: |__|__|

Se tipo de entrevista = 01, seguir para o módulo V1. Caso contrário, encerrar entrevista.

Entrevistador: confirme o endereço do domicílio e preencha:

V1. Endereço confirmado? (V001)
1. Sim 2. Não
Se V1=1, passe ao módulo A. Se V1=2 siga V2.

V2. Mora no mesmo Estado do endereço inicial? (V002)
1. Sim 2. Não
Se V2=1, passe ao módulo A. Se V2=2 siga V3.

V3. Qual Estado de residência atual? (V003)
|__|__| colocar a relação dos 27 estados e retornar no banco os nossos códigos, colocar 98 para país estrangeiro e 99 para ignorado.
Se 98 ou 99 não realizar a entrevista
Caso contrário, siga ao módulo A.

Modulo A - Características dos moradores

Entrevistador leia:<br>*Esta pesquisa tem o objetivo de acompanhar os efeitos do novo coronavírus (COVID-19) na saúde, no trabalho e na vida da população. Suas respostas podem ajudar na busca de soluções para os problemas causados por esta doença.*<br>*Todas as informações prestadas são sigilosas.*

Confirmar as informações:

A1. Nome dos moradores do domicílio:

Número de ordem |___|___| **(A001)**

Nome |___|___|___|___|___|___|___|___|___|___|___|___| **(A0011)**

(Siga A1a)

A1a. Condição no domicílio: (A001A)

1. Pessoa responsável pelo domicílio
2. Cônjuge ou companheiro(a) de sexo diferente
3. Cônjuge ou companheiro(a) do mesmo sexo
4. Filho(a) do responsável e do cônjuge
5. Filho(a) somente do responsável
6. Filho(a) somente do cônjuge
7. Genro ou nora
8. Pai, mãe, padrasto ou madrasta
9. Sogro(a)
10. Neto(a)
11. Bisneto(a)
12. Irmão ou irmã
13. Avô ou avó
14. Outro parente
15. Agregado(a) - Não parente que não compartilha despesas
16. Convivente - Não parente que compartilha despesas
17. Pensionista
18. Empregado(a) doméstico(a)
19. Parente do(a) empregado(a) doméstico(a)

(Siga A1b)

A1b. Data de nascimento:

|_|_|	|_|_|	|_|_|_|_|
Dia	Mês	Ano
**(A001B1)**	**(A001B2)**	**(A001B3)**

(Se a data de nascimento é ignorada, siga A2. Caso contrário, passe ao A3)

A2. Qual sua idade? (A002)
|__|__|__| anos

(Siga A3)

A3. Sexo: (A003)
1. Homem 2. Mulher
(Siga A4)

A4. Cor ou raça: (A004)
1. Branca
2. Preta
3. Amarela
4. Parda
5. Indígena

(Siga A5)

A5. Qual sua escolaridade? (A005)
1. Sem instrução
2. Fundamental incompleto
3. Fundamental completo
4. Médio incompleto
5. Médio completo
6. Superior incompleto
7. Superior completo
8. Pós-graduação, mestrado ou doutorado completo

(Se 6≤A002≤29, siga A6. Caso contrário, passe ao Módulo B)

A6. Se 6≤A002≤16: **Frequenta escola (está matriculado(a))?**
Se 17≤A002≤29: **Frequenta escola ou faculdade (está matriculado(a))? (A006)**
1. Sim 2. Não
(se A6=1, siga A7. Caso contrário, passe ao módulo B)

A7. Na semana passada, _____ foram disponibilizadas ~~teve~~ atividades escolares para realizar em casa (aulas online, deveres, estudo dirigido etc.)? (A007)
1. Sim e realizou pelo menos parte delas
2. Sim, mas não realizou (por qualquer motivo)
3. Não
4. Não, porque estava de férias

(Se A7=1, siga A8. Caso contrário, passe ao módulo B)

A8. Na semana passada, em quantos dias _____ dedicou-se a ~~teve~~ atividades escolares? (A008)
1. 1 dia
2. 2 dias
3. 3 dias
4. 4 dias
5. 5 dias
6. 6 ou 7 dias

(Siga A9)

A9. Na semana passada, quanto tempo por dia ____ gastou fazendo as atividades escolares? (A009)
1. Menos de 1 hora
2. De 1 hora a menos de 2 horas
3. De 2 horas a menos de 5 horas
4. 5 horas ou mais

(passe ao módulo B)

Módulo B – COVID19 – Todos os moradores

Entrevistador leia: Vamos fazer algumas perguntas sobre o novo coronavírus. Primeiro os sintomas. Vou dizer os sintomas e você me responde "sim" ou "não".

B1. Na semana passada, o(a) Sr(a) teve:

a.	Febre	1. Sim	2. Não	3. Não sabe **(B0011)**
b.	Tosse	1. Sim	2. Não	3. Não sabe **(B0012)**
c.	Dor de garganta	1. Sim	2. Não	3. Não sabe **(B0013)**
d.	Dificuldade para respirar	1. Sim	2. Não	3. Não sabe **(B0014)**
e.	Dor de cabeça	1. Sim	2. Não	3. Não sabe **(B0015)**
f.	Dor no peito	1. Sim	2. Não	3. Não sabe **(B0016)**
g.	Náusea/enjoo	1. Sim	2. Não	3. Não sabe **(B0017)**
h.	Nariz entupido ou escorrendo	1. Sim	2. Não	3. Não sabe **(B0018)**
i.	Fadiga/cansaço	1. Sim	2. Não	3. Não sabe **(B0019)**
j.	Dor nos olhos	1. Sim	2. Não	3. Não sabe **(B00110)**
k.	Perda de cheiro ou de sabor	1. Sim	2. Não	3. Não sabe **(B00111)**
l.	Dor muscular/ dor no corpo	1. Sim	2. Não	3. Não sabe **(B00112)**
m.	Diarreia	1. Sim	2. Não	3. Não sabe **(B00113)**

(Se a ou b ou c ou d ou e ou f ou g ou h ou i ou j ou k ou l ou m = 1, siga ao B2. Caso contrário, passe B7.)

Entrevistador leia: Agora vou perguntar sobre o seu comportamento em relação a esses sintomas.

B2. Por causa disso, o(a) Sr(a) foi a algum estabelecimento de saúde (na semana passada)? (B002)

1. Sim 2. Não

(Se B2 = 1, passe ao B4)
(Se B2 = 2, siga B3)
(Se ignorado, passe ao B7)

B3. Que providência(s) o(a) Sr(a) tomou para se recuperar desses sintomas (na semana passada)?

a.	Ficou em casa	1. Sim	2. Não	**(B0031)**
b.	Ligou para algum profissional de saúde	1. Sim	2. Não	**(B0032)**
c.	Tomou remédio por conta própria	1. Sim	2. Não	**(B0033)**
d.	Tomou remédio por orientação médica	1. Sim	2. Não	**(B0034)**
e.	Recebeu visita de algum profissional de saúde do SUS (equipe de saúde da família, agente comunitário, etc.)	1. Sim	2. Não	**(B0035)**
f.	Recebeu visita de profissional de saúde particular	1. Sim	2. Não	**(B0036)**
g.	Outro. Especifique **(B00371)**	1. Sim	2. Não	**(B0037)**

(Passe ao B7)

B4. Em que local(is) buscou o atendimento (na semana passada)?

a.	Posto de saúde/Unidade básica de saúde /Equipe de Saúde da Família (médico, enfermeiro, técnico de enfermagem ou agente comunitário de saúde)	1. Sim	2. Não	**(B0041)**
b.	Pronto socorro do SUS/UPA	1. Sim	2. Não	**(B0042)**
c.	Hospital do SUS	1. Sim	2. Não	**(B0043)**
d.	Ambulatório ou consultório privado ou ligado às forças armadas	1. Sim	2. Não	**(B0044)**
e.	Pronto socorro privado ou ligado às forças armadas	1. Sim	2. Não	**(B0045)**
f.	Hospital privado ou ligado às forças armadas	1. Sim	2. Não	**(B0046)**

(Se b, c, e ou f = 1, siga B5, caso contrário, passe ao B7)

B5. Ao procurar o estabelecimento de saúde, teve que ficar internado(a) por um dia (24 horas) ou mais? (B005)
1. Sim 2. Não 3. Não foi atendido
(Se B5 = 1, siga B6)
(Se B5 = 2 ou 3, passe ao B7)
(Se ignorado, passe ao B7)

B6. Durante esta internação, o(a) Sr(a) foi sedado(a), intubado(a) e colocado(a) em respiração artificial com ventilador? (B006)
1. Sim 2. Não
(Passe ao B7)

B7. O(A) Sr(a) tem algum plano de saúde médico, seja particular, de empresa ou de órgão público? (B007)
1. Sim 2. Não
(Siga B8)

B8. O(A) Sr(a) fez algum teste para saber se estava infectado(a) pelo coronavírus? (B008)
1. Sim
2. Não
(Se B8=1 siga B9A, caso contrário passe ao B10)

B9A. Fez o exame coletado com cotonete na boca e/ou nariz (SWAB)? (B009A)
1. Sim 2.Não
(Se B9A=1, siga B9B, caso contrário, passe ao B9C)

B9B. Qual o resultado? (B009B)
1. Positivo
2. Negativo
3. Inconclusivo
4. Ainda não recebeu o resultado
(Siga B9C)

B9C. Fez o exame de coleta de sangue através de furo no dedo? (B009C)
1. Sim 2.Não
(Se B9C=1, siga B9D, caso contrário, passe ao B9E)

B9D. Qual o resultado? (B009D)
1. Positivo
2. Negativo
3. Inconclusivo
4. Ainda não recebeu o resultado
(Siga B9E)

B9E. Fez o exame de coleta de sangue através da veia do braço? (B009E)
1. Sim 2.Não
(Se B9E=1, siga B9F, caso contrário, passe ao B10)

B9F. Qual o resultado? (B009F)
1. Positivo
2. Negativo
3. Inconclusivo

4. Ainda não recebeu o resultado
(Siga B10)

B10. Algum médico já lhe deu o diagnóstico de alguma dessas doenças?

1. Diabetes (açúcar no sangue)	1. Sim	2. Não	**(B0101)**
2. Hipertensão (pressão alta)	1. Sim	2. Não	**(B0102)**
3. Asma / Bronquite/ Enfisema/ Doença respiratória crônica ou outra doença do pulmão	1. Sim	2. Não	**(B0103)**
4. Doença(s) do coração (infarto, angina, insuficiência cárdica, arritmia, etc)	1. Sim	2. Não	**(B0104)**
5. Depressão	1. Sim	2. Não	**(B0105)**
6. Câncer	1. Sim	2. Não	**(B0106)**

(Siga B11)

B11. Na semana passada, devido à pandemia do Coronavírus, em que medida o(a) Sr(a) restringiu o contato com as pessoas? (B011)

1. Não fez restrição, levou vida normal como antes da pandemia
2. Reduziu o contato com as pessoas, mas continuou saindo de casa para trabalho ou atividades não essenciais e/ou recebendo visitas
3. Ficou em casa e só saiu em caso de necessidade básica (comprar comida, remédio, ir ao médico etc.)
4. Ficou rigorosamente isolado em casa

(Se A002 >=14, passe ao módulo C. Caso contrário, passe ao F6)

Módulo C - Características de trabalho das pessoas de 14 anos ou mais de idade

Entrevistador leia: As próximas perguntas serão sobre trabalho.

C1. Na semana passada, por pelo menos uma hora, o(a) Sr(a) trabalhou ou fez algum bico? (C001)

1. Sim   2. Não

(Se C1 = 1, passe ao C6)
(Se C1 = 2, siga C2)
(Se ignorado, siga C2)

C2. Na semana passada, estava <u>temporariamente</u> afastado de algum trabalho? (C002)

1. Sim   2. Não

(Se C2 = 1, siga C3)
(Se C2 = 2, passe ao C14)
(Se ignorado, passe ao C14)

C3. Qual o principal motivo deste afastamento temporário? (C003)

1. Estava em quarentena, isolamento, distanciamento social ou férias coletivas (siga C4)
2. Férias, folga ou jornada de trabalho variável (passe ao C6)
3. Licença maternidade ou paternidade (passe ao C6)

4. Licença remunerada por motivo de saúde ou acidente da própria pessoa (passe ao C6)
5. Outro tipo de licença remunerada (estudo, casamento, licença prêmio etc.) (passe ao C6)
6. Afastamento do próprio negócio/empresa por motivo de gestação, doença, acidente etc., sem ser remunerado por instituto de previdência (passe ao C5)
7. Fatores ocasionais (mal tempo, paralisação nos serviços de transporte etc.) (passe ao C5)
8. Outro motivo. Especifique. **(C0031)** (passe ao C5)
(Se ignorado, passe ao C14)

C4. O(A) Sr(a) continuou a ser remunerado (mesmo que parcialmente) por esse trabalho? (C005)
1. Sim 2. Não 3. O trabalho já era não remunerado
(Se C4 = 1, passe ao C6. Caso contrário, siga C5)

C5. Há quanto tempo está afastado desse trabalho? (C005)
1. Menos de 1 mês
2. De 1 mês a menos de 1 ano → |__|__| meses **(C0051)**
3. De 1 ano a menos de 2 anos → |_1_| ano e |__|__| meses **(C0052)**
4. 2 anos ou mais → |__|__| anos **(C0053)**

(Se C5 = 1) ou (C5 = 2, sendo C0051= 01, 02 ou 03), siga C6. Caso contrário, passe ao C14)

C6. O(A) Sr(a) tem mais de um trabalho? (C006)
1. Sim 2. Não
(Siga C7)

C7. No trabalho (único ou principal) que tinha nessa semana, o(a) Sr(a) era: (C007)
1. Trabalhador doméstico (empregado doméstico, cuidador, babá etc.)
2. Militar do exército, da marinha, da aeronáutica
3. Policial militar ou bombeiro militar
4. Empregado do setor privado
5. Empregado do setor público (inclusive empresas de economia mista)
6. Empregador (que tem ao menos um empregado, formalizado ou não)
7. Conta própria (sem empregado, podendo trabalhar com sócio ou com ajuda de parente não remunerado)
8. Trabalhador não remunerado em ajuda a morador do domicílio ou parente
9. Estava fora do mercado de trabalho (fazia apenas afazeres domésticos, cuidados de pessoas, produção para próprio consumo ou trabalho voluntário)

(Se C7 = 9, passe C14.
Se C7 = 2, impute C7a=1 e passe ao C8.
Se C7 = 3, impute C7a=2 e passe ao C8.
Se C7 = 1 ou 4, passe a C7b.
Se C7 = 5, passe ao C7a.
Caso contrário, passe ao C7c)

C7a. Esse trabalho (único ou principal) era na área: (C007A)
1. Federal 2. Estadual 3. Municipal
(Siga C7b)

C7b. Se C7=1 ou 4: **Tem carteira de trabalho assinada?**
Se C7=5**: Tem carteira de trabalho assinada ou é servidor público estatutário (federal, estadual ou municipal) neste trabalho (único ou principal)? (C007B)**
1. Sim, tem carteira de trabalho assinada
2. Sim, é servidor público estatutário *(inibir visualização se C7=1 ou 4)*
3. Não

(Siga C7c)

C7c. Que tipo de trabalho, cargo ou função você realiza no seu trabalho (único ou principal)? (C007C)

1. Empregado doméstico, diarista, cozinheiro (em domicílios particulares)
2. Faxineiro, auxiliar de limpeza etc. (em empresa pública ou privada)
3. Auxiliar de escritório, escriturário
4. Secretária, recepcionista
5. Operador de Telemarketing
6. Comerciante (dono do bar, da loja etc.)
7. Balconista, vendedor de loja
8. Vendedor a domicílio, representante de vendas, vendedor de catálogo (Avon, Natura etc.)
9. Vendedor ambulante (feirante, camelô, comerciante de rua, quiosque)
10. Cozinheiro e garçom (de restaurantes, empresas)
11. Padeiro, açougueiro e doceiro
12. Agricultor, criador de animais, pescador, silvicultor e jardineiro
13. Auxiliar da agropecuária (colhedor de frutas, boia fria, etc.)
14. Motorista (de aplicativo, de taxi, de van, de mototáxi, de ônibus)
15. Motorista de caminhão (caminhoneiro)
16. Motoboy
17. Entregador de mercadorias (de restaurante, de farmácia, de loja, Uber Eats, IFood, Rappy etc.)
18. Pedreiro, servente de pedreiro, pintor, eletricista, marceneiro
19. Mecânico de veículos, máquinas industriais etc.
20. Artesão, costureiro e sapateiro
21. Cabeleireiro, manicure e afins
22. Operador de máquinas, montador na indústria
23. Auxiliar de produção, de carga e descarga
24. Professor da educação infantil, de ensino fundamental, médio ou superior
25. Pedagogo, professor de idiomas, música, arte e reforço escolar
26. Médico, enfermeiro, profissionais de saúde de nível superior
27. Técnico, profissional da saúde de nível médio
28. Cuidador de crianças, doentes ou idosos
29. Segurança, vigilante, outro trabalhador dos serviços de proteção
30. Policial civil
31. Porteiro, zelador
32. Artista, religioso (padre, pastor etc.)
33. Diretor, gerente, cargo político ou comissionado
34. Outra profissão de nível superior (advogado, engenheiro, contador, jornalista etc.)
35. Outro técnico ou profissional de nível médio
36. Outros (especifique) **(C007C1)**

(Se C7= 1 e C3=1 e C7b=1, impute C7d = 24 e passe ao C7f.
Se C7=1 e (C3^=1 ou C7b^=1), impute C7d = 24 e passe ao C8.
Caso contrário, Siga C7d)

C7d. Qual é a principal atividade do negócio ou empresa em que você trabalha? (C007D)

1. Agricultura, pecuária, produção florestal e pesca
2. Extração de petróleo, carvão mineral, minerais metálicos, pedra, areia, sal etc.
3. Indústria da transformação (inclusive confecção e fabricação caseira)
4. Fornecimento de eletricidade e gás, água, esgoto e coleta de lixo
5. Construção
6. Comércio no atacado e varejo;
7. Reparação de veículos automotores e motocicletas

8. Transporte de passageiros
9. Transporte de mercadorias
10. Armazenamento, correios e serviços de entregas
11. Hospedagem (hotéis, pousadas etc.)
12. Serviço de alimentação (bares, restaurantes, ambulantes de alimentação)
13. Informação e comunicação (jornais, rádio e televisão, telecomunicações e informática)
14. Bancos, atividades financeiras e de seguros
15. Atividades imobiliárias
16. Escritórios de advocacia, engenharia, publicidade e veterinária (Atividades profissionais, científicas e técnicas)
17. Atividades de locação de mão de obra, segurança, limpeza, paisagismo e teleatendimento
18. Administração pública (governo federal, estadual e municipal)
19. Educação
20. Saúde humana e assistência social
21. Organizações religiosas, sindicatos e associações
22. Atividade artísticas, esportivas e de recreação
23. Cabeleireiros, tratamento de beleza e serviços pessoais
24. Serviço doméstico remunerado (*será imputado da posição na ocupação*)
25. Outro (especifique) **(C007D1)**

(Se C7 = 6, siga C7e.
Se C3=1 e C7 = 4 e C7b=1, passe ao C7f.
Caso contrário, passe ao C8)

C7e. Na semana passada, quantos empregados trabalhavam nesse negócio/empresa que ___ tinha? (C007E)

1. 1 a 5 empregados → |__| **(C007E1)**
2. 6 a 10 empregados → |__|__| **(C007E2)**
3. 11 ou mais empregados

(Passe ao C8)

C7f. No trabalho (único ou principal) que tinha nessa semana, o(a) Sr(a) estava com o contrato de trabalho suspenso? (C007F)

1. Sim 2. Não

(Siga C8.)

C8. Quantas horas, por semana, o(a) Sr(a) <u>normalmente</u> trabalha (em todos os trabalhos)? (C008)

|___|___|___| horas

(Se C2=1, imputar "0" em C9 e passe ao C10. Caso contrário, siga C9.)

C9. Quantas horas, na semana passada, o(a) Sr(a), de fato, trabalhou (em todos os trabalhos)? (C009)

|___|___|___| horas

Se C009<40, siga C9a
Caso contrário:
(Se C6=2 e C7= 1 a 7, siga C10)
(Se C1=1 e C6=2 e C7= 8 impute (C010=1 e C0104=4) e passe ao C12)
(Se C2=1 e C6=2 e C7= 8 impute (C010=1 e C0104=4) e passe ao C14)
(Se C6=1, siga C10)
(Se C9=ignorado, passe ao C10)

C9a. Na semana passada, o(a) Sr(a) gostaria de ter trabalhado mais horas do que as de fato trabalhadas? (C009A)

1. Sim 2. Não

(Se C6=2 e C7= 1 a 7, siga C10)
(Se C1=1 e C6=2 e C7= 8 impute (C010=1 e C0104=4) e passe ao C12)
(Se C2=1 e C6=2 e C7= 8 impute (C010=1 e C0104=4) e passe ao C14)
(Se C6=1, siga C10)
(Se C9=ignorado, passe ao C10)

C10. Se C6=2: **Quanto recebe (ou retira) normalmente em seu trabalho? (C010)**
Se C6=1**: Quanto recebe (ou retira) normalmente em todos os seus trabalhos?**

1. Valor em dinheiro **(C0101)**
   Faixa **(C01011)** (R$): |___|___|___|___|___|___|___|___| ,00 **(C01012)**

2. Valor estimado em produtos e mercadorias **(C0102)**
   Faixa **(C01021)** (R$): |___|___|___|___|___|___|___|___| ,00 **(C01022)**
3. Somente em benefícios **(C0103)**
4. Não remunerado **(C0104)**

(Se C10 = 1 ou 2, siga C11a)
(Se C10 = 3 ou 4 e C1=1 e C7=1 e C7b=3 impute C12=1 e passe ao C14)
(Se C10 = 3 ou 4 e C1=1 e C7=2 ou 3 ou 4 ou 5 ou 8, passe ao C12)
(Se C10 = 3 ou 4 e C2=1 e C7=1, 4, 5 e C7b=3, passe ao C14)
(Se C10 = 3 ou 4 e C2=1 e C7=2 ou 3, encerra o módulo e passe ao módulo D)
(Se C10 = 3 ou 4 e C2=1 e C7= 8, passe ao C14)
(Se C10 = 3 ou 4 e C1=1 e C7=1 e C7b=ignorado, impute C12=1, encerra o módulo e passe ao módulo D)
(Se C10 = ignorado e C1=1 e C7=1 e 1≤C7b≤2, impute C12=1, encerra o módulo e passe ao módulo D)
(Se C10 = ignorado e C1=1 e C7=1 e C7b= ignorado, impute C12=1, encerra o módulo e passe ao módulo D)
(Se C10 = ignorado e C1=1 e C7=1 e C7b=3 impute C12=1 e passe ao C14)
(Se C10 = ignorado e C1=1 e C7=2 ou 3 ou 4 ou 5 ou 6 ou 7 ou 8, passe ao C12)
(Se C10 = ignorado e C1=1 e C7= ignorado, passe ao C12)
(Se C10 = ignorado e C2=1 e C7=1, 4, 5 e C7b=3, passe ao C14)
(Se C10 = ignorado e C2=1 e C7= 6, 7 e 8, passe ao C14)
(Caso contrário, encerra o módulo e passe ao módulo D)

C11a. Se C6=2: **Quanto recebeu (retirou), de fato, em seu trabalho, em (mês de referência)? (C011A)**
Se C6=1**: Quanto recebeu (retirou), de fato, em todos os seus trabalhos, em (mês de referência)?**

1. Valor em dinheiro **(C011A1)**
   Faixa **(C011A11)** (R$):|___|___|___|___|___|___|___|___|,00 **(C011A12)**

2. Valor estimado em produtos e mercadorias **(C011A2)**
   Faixa **(C011A21)** (R$): |___|___|___|___|___|___|___|___| ,00 **(C011A22)**

(Se C2=1 e C7=1, 4, 5 e C7b=3, passe ao C14)
(Se C2=1 e C7= 6, 7 e 8, passe ao C14)
(Se C2=1 e C7=1, 4, 5 e 1≤C7b≤2, encerra o módulo e passe ao módulo D)
(Se C2=1 e C7=2 ou 3, encerra o módulo e passe ao módulo D)
(Se C2=1 e C7=1, 4, 5 e C7b=ignorado, encerra o módulo e passe ao módulo D)
(Se C1=1 e C7=1 e 1≤C7b≤2, impute C12=1, encerra o módulo e passe ao módulo D)
(Se C1=1 e C7=1 e C7b=ignorado, impute C12=1, encerra o módulo e passe ao módulo D)

(Se C1=1 e C7=1 e C7b=3 impute C12=1 e passe ao C14)
(Se C2=1 e C7=ignorado, encerra o módulo e passe ao módulo D)
(Caso contrário siga C12.)

C12. Na maior parte do tempo, na semana passada, esse trabalho (único ou principal) foi exercido no mesmo local em que costuma trabalhar? (C012)

1. Sim 2. Não

(Se C12=1 e C7=1, 4, 5 e C7b=3, passe ao C14)
(Se C12=1 e C7= 6, 7 e 8, passe ao C14)
(Se C12=1 e C7=1, 4, 5 e 1≤C7b≤2, encerra o módulo e passe ao módulo D)
(Se C12=1 e C7=2 ou 3, encerra o módulo e passe ao módulo D)
(Se C12=1 e C7=ignorado, encerra o módulo e passe ao módulo D)
(Se C12=2, siga C13)
(Se C12=ignorado e C7=1, 4, 5 e C7b=3, passe ao C14)
(Se C12=ignorado e C7= 6, 7 e 8, passe ao C14)
(Se C12= ignorado e C7=1, 4, 5 e 1≤C7b≤2, encerra o módulo e passe ao módulo D)
(Se C12= ignorado e C7=2 ou 3, encerra o módulo e passe ao módulo D)
(Se C12=ignorado e C7=ignorado, encerra o módulo e passe ao módulo D)
(Caso contrário, encerra o módulo e passe ao módulo D)

C13. Na semana passada, o(a) Sr(a) estava em trabalho remoto (home office ou teletrabalho)? (C013)
(Obs. Mesmo que parcialmente)

1. Sim 2. Não

(Se C7=1, 4, 5 e C7b=3, passe ao C14)
(Se C7= 6, 7 e 8, passe ao C14)
(Se C7=1, 4, 5 e 1≤C7b≤2, encerra o módulo e passe ao módulo D)
(Se C7=2 ou 3, encerra o módulo e passe ao módulo D)
(Se C13 = ignorado e C7=1, 4, 5 e C7b=3, passe ao C14)
(Se C13 = ignorado e C7= 6, 7 e 8, passe ao C14)
(Se C13= ignorado e C7=1, 4, 5 e 1≤C7b≤2, encerra o módulo e passe ao módulo D)
(Se C13= ignorado e C7=2 ou 3, encerra o módulo e passe ao módulo D)
(Se C13 = ignorado e C7=ignorado, encerra o módulo e passe ao módulo D)
(Se C7=ignorado, encerra o módulo e passe ao módulo D)
(Caso contrário, encerra o módulo e passe ao módulo D)

C14. O(A) Sr(a) contribui para o INSS? (C014)

1. Sim 2. Não

(Se C2=2 ou ((C5=2 e C0051>=4 meses) ou C5 = 3 ou C5 = 4) ou C7 = 9, siga C15)
(Se C14= ignorado e ((C2=2 ou (C5=2 e C0051>=4 meses) ou C5 = 3 ou C5 = 4)) ou C7 = 9), siga C15)
(Caso contrário, encerra o módulo e passe ao módulo D)

C15. Na semana passada, ___ tomou alguma providência efetiva para conseguir trabalho? (C015)

1. Sim 2. Não

(Se C15 = 2, siga C16)
(Se C15 = 1, encerra o módulo e passe ao módulo D)
(Se C15 = ignorado, encerra o módulo e passe ao módulo D)

C16. Qual o principal motivo de não ter procurado trabalho na semana passada? (C016)

1. Devido à pandemia (isolamento, quarentena ou distanciamento social)
2. Por problemas de saúde ou gravidez

3. Estava estudando
4. Não quer trabalhar ou é aposentado
5. Não tinha experiência ou qualificação
6. Acha que não vai encontrar trabalho por ser muito jovem ou idoso
7. Não havia trabalho na localidade
8. Tinha que cuidar dos afazeres domésticos e/ou de parentes
9. Estava aguardando resposta de medida tomada para conseguir trabalho
10. Outro motivo. Especifique. **(C0161)**

(Se C16=4, impute C17a = 2 e passe ao módulo D.
(Se C16=9, impute C17a = 1 e passe ao módulo D.
Caso contrário, siga C17a)

C17a. Embora você não tenha procurado trabalho, gostaria de ter trabalhado na semana passada? (C017A)

1. Sim 2. Não

(Encerra o módulo. Passe ao módulo D)

Módulo D - Rendimentos de outras fontes dos moradores de 14 anos ou mais de idade

D1. (Se mais de um morador) No seu domicilio, em (mês de referência), alguém, incluindo o(a) Sr(a), recebeu rendimento de:
(Se apenas um morador) Em (mês de referência), o(a) Sr(a) recebeu rendimento de:
(Observação: se houver mais de um morador recebendo o rendimento, some todos os valores recebidos deste tipo de rendimento)

a. aposentadoria ou pensão? (D0011)
1. Sim R$ _________________ (valor mensal) **(D0013)** Faixa**: (D0012)**
2. Não

b. pensão alimentícia, doação ou mesada em dinheiro de pessoa que não morava no domicílio? (D0021)
1. Sim R$ _________________ (valor mensal) **(D0023)** Faixa**: (D0022)**
2. Não

c. Bolsa família (D0031)
1. Sim R$ _________________ (valor mensal) **(D0033)** Faixa**: (D0032)**
2. Não

d. Benefício Assistencial de Prestação Continuada – BPC-LOAS? (D0041)
1. Sim R$ _________________ (valor mensal) **(D0043)** Faixa**: (D0042)**
2. Não

e. Auxílios emergenciais relacionados ao *coronavíus*? (D0051)
1. Sim R$ _________________ (valor mensal) **(D0053)** Faixa**: (D0052)**
2. Não

f. Seguro desemprego ou seguro-defeso? (D0061)
1. Sim R$ _________________ (valor mensal) **(D0063)** Faixa**: (D0062)**

2. Não

g. Outros rendimentos, como aluguel, arrendamento, previdência privada, bolsa de estudos, rendimentos de aplicação financeira etc.? (D0071)
1. Sim R$ ________________ (valor mensal) **(D0073)** Faixa**: (D0072)**
(Especifique) (D0074)
2. Não
(Passe ao módulo E)

Módulo E – Empréstimos

E1. Durante o período da pandemia alguém deste domicílio solicitou algum empréstimo? (E001)
1. Sim, e pelo menos um morador conseguiu 2. Sim, mas nenhum morador conseguiu
3. Não solicitou
(Se E1=1, siga E2. Caso contrário, passe ao módulo F)

E2. Este empréstimo foi/foram adquirido(s) com:

1.	Banco ou financeira	1. Sim	2. Não	**(E0021)**
2.	Parente ou amigo	1. Sim	2. Não	**(E0022)**
3.	Empregador, patrão	1. Sim	2. Não	**(E0023)**
4.	Outro local ou pessoa. Especifique: _____ **(E00241)**	1. Sim	2. Não	**(E0024)**

(Passe ao módulo F)

Módulo F – Domicílio, propriedade e valor do aluguel

F1. Este domicílio é: (F001)
1. Próprio de algum morador - já pago
2. Próprio de algum morador - ainda pagando
3. Alugado
4. Cedido por empregador
5. Cedido por familiar
6. Cedido de outra forma
7. Outra condição. Especifique: _____ **(F0011)**

(Se F1 = 3, siga F2)
(Caso contrário, passe ao F2a)

F2. Qual foi o valor mensal do aluguel pago, ou que deveria ter sido pago, no mês de ___ (mês de referência)?
(R$): |__|__|__|__|__|__|__|__| **(F0021)** Faixa de valores **(F0022)**
(Siga F2a)

F2a. No seu domicílio há os seguintes itens básicos de limpeza e proteção:

a)	Sabão ou detergente	1. Sim	2. Não	3. Não sabe **(F002A1)**
b)	Álcool 70% ou superior (em gel ou líquido)	1. Sim	2. Não	3. Não sabe **(F002A2)**
c)	Máscaras	1. Sim	2. Não	3. Não sabe **(F002A3)**
d)	Luvas descartáveis	1. Sim	2. Não	3. Não sabe **(F002A4)**
e)	Água sanitária ou desinfetante	1. Sim	2. Não	3. Não sabe **(F002A5)**

(Siga F6)

F6. Quem respondeu ao questionário? (F0061)

1. Pessoa moradora |__|__| **(F006)** (*Abrir relação de moradores para escolher o nome e carrega o número de ordem)*
2. Pessoa não moradora

(Para preenchimento pelo entrevistador ao final da entrevista) tipo de entrevista

**Código**	**Tipo de entrevista**
	**Entrevista concluída com sucesso**
01	Realizada
	**Não foi possível estabelecer contato com o domicílio**
02	Domicílio não pode ser contactado após várias tentativas
	**Domicílio (ou telefone) contactado sem entrevista completa**
03	Recusa
04	Telefone não corresponde ao domicílio selecionado
05	Respondente informa que telefone é de unidade não residencial
06	Respondente é criança (menor de 14 anos) e não há adulto presente
07	Interrompida sem condições de completar
08	Outro motivo
