# Supplementary material for: Social inequalities in self-reported SARS-CoV-2 infection in Brazilian adults: PNAD COVID-19
Source: Rev Bras Epidemiol. 2024 Aug 30;27:e240042. doi: 10.1590/1980-549720240042 (PMC11383518; doi:10.1590/1980-549720240042)
Supplement: Translation

# Translation: Desigualdades sociais no autorrelato de infecção por SARS-CoV-2 em adultos brasileiros: Pnad COVID-19

**DOI:** 10.1590/1980-549720240042.2

**Authors:** Mateus Andrade Rocha, Cândido Norberto Bronzoni de Mattos, Marcos Pascoal Pattussi

**Affiliations:** I Universidade do Vale do Rio dos Sinos, Programa de Pós-Graduação em Saúde Coletiva – São Leopoldo (RS), Brasil.; II Universidade Federal de Santa Catarina, Programa de Pós-Graduação em Odontologia – Florianópolis (SC), Brasil.

**Keywords:** Saúde pública, COVID-19, Iniquidade social, Adultos

## Abstract

**Objetivo:**

Investigar as desigualdades relacionadas a raça/etnia e condição socioeconômica no autorrelato de resultado positivo para COVID-19 em adultos brasileiros.

**Métodos:**

Os dados disponibilizados pela Pesquisa Nacional por Amostra de Domicílios (Pnad) COVID-19 (julho/setembro/novembro, 2020) foram utilizados nesta investigação retrospectiva. As análises consideraram o desenho amostral, unidades primárias de amostragem, estratos e pesos amostrais. Regressão de Poisson com variância robusta foi utilizada para estimar as razões de prevalência (RP) e o intervalo de confiança de 95% (IC95%) das associações.

**Resultados:**

Nos meses de julho, setembro e novembro de 2020, referente ao teste rápido, os indígenas tinham 2,45 (IC95% 1,48–4,08), 2,53 (IC95% 1,74–4,41) e 1,23 (IC95% 1,11–1,86) vezes maior probabilidade de reportar o histórico positivo de infecção por SARS-CoV-2, respectivamente. Com relação ao teste RT-PCR no mês de novembro, os indígenas apresentaram mais chance de testarem positivo para COVID-19 (RP: 1,90; IC95% 1,07–3,38). Foi observado que o grupo de indígenas apresentou 1,86 (IC95% 1,05–3,29) e 2,11 (IC95% 1,12–3,59) vezes mais chances de positivarem para COVID-19 em setembro e novembro (2020). A renda esteve associada com a testagem positiva para a COVID-19: no mês de novembro, indivíduos com renda variando entre R$ 0,00–R$ 1,044 tiveram maior probabilidade (RP: 1,69; IC95% 1,16–23,06) de testarem positivo através do teste RT-PCR; participantes com renda variando na referida faixa de valor também apresentaram maior chance de serem diagnosticados com COVID-19 através de testes sanguíneos (RP: 1,72; IC95% 1,43–2,07).

**Conclusão:**

Os dados apresentados evidenciam a associação entre a raça/etnia e o *status* econômico com o resultado positivo para COVID-19.

## INTRODUÇÃO

No primeiro trimestre de 2020, um novo tipo de coronavírus da COVID-19 altamente transmissível e patogênico foi responsável por infectar um elevado número de indivíduos globalmente, desencadeando a pandemia de COVID-19^1^. O vírus da COVID-19 afeta o sistema respiratório, causando sintomas leves em muitas pessoas, mas pode levar a condições críticas em uma porcentagem dos casos, com danos alveolares maciços e insuficiência respiratória que podem contribuir para a morte^2,3^. O Brasil emergiu como epicentro pandêmico da doença do coronavírus. Somente durante a primeira onda da pandemia (de março a novembro de 2020), foram registrados mais de 7,9 milhões de casos e mais de 100 mil mortes pela doença no país^4^. O diagnóstico da infecção pode ser realizado através de uma variedade de testes com fluídos orais e sanguíneos. Contudo o método diagnóstico padrão-ouro para COVID-19 se baseia em um teste molecular da reação em cadeia de polimerase de transcrição reversa RT-PCR, com o objetivo de detectar o RNA do vírus em amostras respiratórias como cotonetes nasofaríngeos ou aspirado brônquico^5^.

Evidências demonstram que o sexo masculino, a idade mais avançada, hábitos não saudáveis (exemplo, fumar tabaco), a obesidade e o diagnóstico de doenças crônicas (exemplo, hipertensão, diabetes e doenças respiratórias e cardiovasculares) apresentam maior risco de infecção e evolução para um estado crítico ou mortal da doença^6–9^. Além dos fatores relacionados às condições orgânicas individuais, os riscos de infecção e o curso grave estão distribuídos de forma desigual na sociedade^4^. A literatura internacional, especialmente em países desenvolvidos, relata um maior impacto da doença nas pessoas de baixo nível socioeconômico e em grupos minoritários raciais/étnicos^10,11^.

Recentemente, estudo brasileiro de base populacional constatou que as populações indígenas, famílias numerosas e famílias com baixa condição socioeconômica possuíam uma prevalência mais elevada de anticorpos para o SARS-CoV- 2 em relação à população branca, famílias não numerosas e com alto nível socioeconômico^12^. Achados transversais também evidenciaram que a menor escolaridade e renda e o maior número de indivíduos na residência familiar estavam fortemente associados com taxas mais elevadas de mortalidade por COVID-19^13^. Nesse sentido, o objetivo deste estudo foi investigar as desigualdades de raça e renda mensal no autorrelato de infecção por SARS-CoV-2 em adultos durante a primeira onda da pandemia no Brasil.

## MÉTODOS

Este estudo retrospectivo foi realizado com os dados disponibilizados pela Pesquisa Nacional por Amostra de Domicílios (Pnad) COVID-19. O objetivo da pesquisa era estimar o número de pessoas com sintomas referidos associados à síndrome gripal e monitorar os impactos da pandemia de COVID-19 no mercado de trabalho brasileiro^14^. O reporte deste estudo foi realizado de acordo com as orientações do *Strengthening the Reporting of Observational Studies in Epidemiology* (Strobe) *Statement* ^15^. A utilização de dados secundários públicos neste estudo dispensa a aprovação do uso dessas informações pelo Comitê de Ética em Pesquisa.

Os dados foram coletados por cerca de 2 mil agentes do Instituto Brasileiro de Geografia e Estatística (IBGE) com base em entrevistas estruturadas via telefone em cerca de 193,6 mil domicílios, distribuídos em 3.364 municípios em todas as macroregiões do país. A coleta de dados ocorreu entre os meses de maio e novembro de 2020, no curso da pandemia da COVID-19 no Brasil^12^. Para este estudo, foram utilizados os dados dos meses de julho, setembro e novembro de 2020.

A seleção e treinamento da equipe de pesquisa ocorreu por meio da Coordenação de Treinamento e Aperfeiçoamento da Escola Nacional de Ciências e Estatística do IBGE.. O treinamento foi composto por dois módulos de conteúdo, um deles referente à abordagem ao participante no contato telefônico e o outro sobre a aplicação do questionário da pesquisa. O processo metodológico completo do Pnad COVID-19 pode ser acessado em estudos prévios^14,16,17^.

Os participantes foram convidados a responder às seguintes perguntas:

1. "*Você já foi diagnosticado com o vírus da COVID-19?*" (sim; não; não sei responder);
2. *"Você já realizou algum teste para saber se estava infectado pelo coronavírus?"* (sim; não; não sei responder);
3. "*Qual foi o teste realizado para verificar se você estava com COVID-19?"* (coleta com cotonete na boca e(ou) nariz RT-PCR; através de furo no dedo (teste rápido); coleta de sangue através da veia do braço (testagem sanguínea).

Respostas inconclusivas, sem resultado ou resultados ignorados nos testes foram excluídos. Os históricos de testagem positiva (sim ou não) nos testes utilizadas foram considerados os desfechos deste estudo.

Características relacionadas a raça/etnia e renda familiar dos indivíduos também foram obtidas. A renda familiar mensal dos participantes foi coletada em número absoluto (real brasileiro – R$) e posteriormente classificada de acordo com a distribuição pelos quartis, sendo: R$≥R$ 2.500; R$ 2.499–R$ 1.430; R$ 1.429–R$ 1.045; R$1.044–0. A raça/etnia dos participantes foi identificada de acordo com os critérios do IBGE e considerou participantes brancos, pretos, amarelos, pardos e indígenas. Outras variáveis de exposição exploraram os aspectos sociodemográficos, relacionados ao distanciamento social e à posse de itens de limpeza e proteção também foram considerados como fatores de confusão do presente estudo: idade em anos completos (categorizada em faixa-etária com base nos critérios do IBGE: 18–29; 30–39; 40–49; 50–59; ≥60 anos), sexo (masculino; feminino), escolaridade (ensino superior completo/incompleto; ensino médio completo; ensino fundamental completo/incompleto; ensino fundamental incompleto/sem instrução), morbidades (sem morbidades; 1 morbidade; 2 morbidades; ≥3 morbidades), utensílios álcool (não possui álcool; possui álcool), utensílios máscara (não possui máscara; possui máscara), distanciamento (rigorosamente isolado; sair apenas para necessidades básicas; sair para trabalho ou atividades essenciais; não fez distanciamento). O questionário de pesquisa contendo as variáveis utilizadas neste estudo podem ser encontradas no Apêndice 1.

Todas as análises estatísticas foram realizadas usando Stata Statistical Package (Versão 16.0) (Stata Corp, College Station, Texas, EUA). Todos os dados que suportam os achados deste estudo estão disponíveis com o autor correspondente mediante solicitação prévia, de acordo com os Princípios de Dados FAIR (www.force11.org/group/fairgroup/fairprinciples). As variáveis foram descritas através das frequências relativas e absolutas. Regressão de Poisson com variância robusta foi utilizada para estimar a razão de prevalência (RP) e o intervalo de confiança de 95% (IC95%) na associação dos desfechos e as variáveis de exposição. Na análise multivariada, as variáveis de exposição foram controladas por fatores de confusão associados ao desfecho num nível de significância menor que 10%. As análises levaram em conta o desenho amostral, unidades primárias de amostragem, estratos e pesos amostrais. Um nível de significância menor que 5% foi adotado para considerar as associações entre exposição e desfecho como significativas.

## RESULTADOS

De maneira geral, a realização de algum teste para COVID-19 foi reportada por 26,673 (julho), 35,587 (setembro) e 45,180 (novembro) participantes. A realização do teste de RT-PCR para COVID-19 nos meses de julho, setembro e novembro foi reportada por 7,026, 12,943 e 18,308 participantes, respectivamente. Nesse mesmo período, 49,407 testagens rápidas foram realizadas, em que: julho: n=11,630; setembro: n=16,954; novembro: n=20,823. Ainda, o histórico de testagem sanguínea foi reportado por 6,886 (julho), 10,668 (setembro) e 13,102 (novembro) indivíduos entrevistados. As Tabelas 1 e 2 apresentam a distribuição amostral das variáveis de exposição de acordo com o histórico de infecção por SARS-CoV-2 por modalidades de testagem.

**Tabela 1:** Descrição da amostra conforme variáveis demográficas e socioeconômicas de acordo com a prevalência de teste RT-PCR positivo em julho (n=7.026), setembro (n=12.943) e novembro (n=18.308) e teste rápido para COVID-19 em julho (n=11.630), setembro (n=16.954) e novembro (n=20.823), 2020.

Variáveis		Julho		Setembro		Novembro	
		n (%)	+COVID (IC95%)*	n (%)	+COVID (IC95%)*	n (%)	+COVID (IC95%)*
RT-PCR							
	Total	7.026 (100)	27,8 (26,0–29,6)	12.943 (100)	28,3 (27,8–28,7)	18.308 (100)	29,3 (28,9–29,7)
Raça							
	Branca	3.320 (47,3)	26,6 (24,3–29,0)	6.245 (48,3)	26,9 (26,4–27,3)	9.201 (50,3)	28,1 (26,2–30,0)
	Preta	601 (8,6)	27,8 (23,1–33,0)	1.139 (8,8)	27,5 (27,0–28,1)	1.570 (8,6)	27,3 (23,7–30,8)
	Parda	3.028 (43,1)	29,4 (26,9–32,1)	5.420 (41,9)	30,7 (28,6–32,8)	7.361 (40,2)	31,7 (29,9–33,4)
	Amarela	52 (0,7)	22,7 (7,8–50,4)	98 (0,8)	14,4 (9,6–199,2)	120 (0,7)	20,0 (15,4–24,6)
	Indígena	22 (0,3)	35,8 (13,8–66,0)	38 (0,3)	39,5 (31,6–47,4)	52 (0,3)	40,0 (34,5–46,3)
Faixa etária (anos)							
	18–29	1.317 (20,3)	24,4 (21,4–27,8)	2.722 (21,0)	26,3 (22,7–29,8)	3.923 (21,4)	27,1 (25,2–28,9)
	30–39	1.711 (26,3)	29,1 (26,0–32,3)	3.126 (24,2)	29,5 (26,9–32,1)	4.355 (23,8)	31,2 (29,4–32,9)
	40–49	1.443 (22,2)	29,2 (26,1–32,6)	2.847 (22,0)	29,4 (26,3–325)	3.953 (21,6)	30,8 (28,5–33,1)
	50–59	1.065 (16,4)	32,4 (28,5–36,5)	2.173 (16,8)	29,3 (25,4–33,2)	3.018 (16,5	31,2 (28,9–33,5)
	≥60	959 (14,8)	26,1 (22,2–30,4)	2.075 (16,0)	27,6 (23,6–31,6)	3.059 (16,7)	26,5 (24,4–28,6)
Sexo							
	Masculino	3.240 (46,1)	27,7 (25,6–30,0)	5.852 (45,2)	28,7 (25,3–31,3)	8.260 (45,1)	29,7 (27,1–32,4)
	Feminino	3.786 (53,9)	27,8 (25,7–29,9)	7.091 (54,8)	28,3 (26,8–29,7)	10.048 (54,9)	29,3 (28,1–30,5)
Escolaridade							
	Superior	2.885 (41,1)	25,9 (23,7–28,3)	5.419 (41,9)	27,0 (23,8–30,2)	7.899 (43,1)	29,1 (27,1–31,0)
	EM completo	1.957 (27,8)	31,6 (28,7–34,6)	3.891 (30,1)	30,6 (26,5–34,7)	5.427 (29,6)	30,9 (28,2–33,6)
	EF completo/EM incompleto	822 (11,7)	29,9 (25,5–34,6)	1.567 (12,1)	28,9 (23,6–34,1)	2.166 (11,8)	29,8 (27,1–32,5)
	EF incompleto/sem instrução	1.362 (19,4)	25,1 (21,6–28,9)	2.066 (16,0)	27,9 (23,6–32,1)	2.816 (15,4)	27,6 (24,7–30,5)
Renda							
	≥R$ 2.500	1.993 (43,7)	28,1 (25,3–31,1)	3.610 (41,0)	27,3 (24,3–30,3)	5.055 (41,0)	30,0 (27,8–32,1)
	R$ 2.499–R$ 1.430	1.183 (25,9)	27,6 (24,4–31,1)	2.292 (26,1)	28,9 (25,6–32,1)	3.189 (25,9)	29,4 (26,2–32,6)
	R$ 1.429–R$ 1.045	1.081 (23,7)	27,6 (24,2–31,3)	2.212 (25,1)	29,6 (26,1–33,1)	3.103 (25,2)	30,6 (28,2–33,1)
	R$ 1.044–0	307 (6,7)	25,3 (18,7–33,3)	682 (7,8)	26,7 (20,3–33,1)	968 (7,9)	27,7 (21,9–33,4)
Teste rápido							
	Total	11.630 (100)	16,2 (15,1–17,3)	16.954 (100)	19,7 (18,5–20,8)	20.823 (100)	19,9 (18,7–21,8)
Raça							
	Branca	4.707 (40,5)	13,1 (11,8–14,6)	7.033 (41,5)	16,4 (13,9–18,8)	8.771 (42,1)	17,0 (14,9–19,0)
	Preta	1.035 (8,9)	16,3 (13,1–20,1)	1.622 (9,6)	20,4 (17,3–23,5)	1.962 (9,4)	19,3 (16,7–21,9)
	Parda	5.748 (49,4)	18,9 (17,3–20,6)	8.108 (47,8)	22,3 (20,6–24,1)	9.856 (47,3)	22,2 (20,9–23,5)
	Amarela	75 (0,7)	12,7 (5,7–26,1)	86 (0,5)	18,6 (4,6–32,6)	117 (0,6)	18,8 (3,8–33,8)
	Indígena	61 (0,5)	34,9 (20,9–52,2)	101 (0,6)	36,6 (22,8–50,4)	113 (0,5)	22,1 (11,1–33,1)
Faixa etária (anos)							
	18–29	2.085 (19,5)	15,2 (13,2–17,4)	3.418 (20,2)	19,7 (17,2–22,2)	4.127 (19,8)	19,8 (17,9–21,7)
	30–39	2.676 (25,0)	15,9 (14,0–17,9)	3.977 (23,5)	19,2 (17,0–21,4)	4.716 (22,6)	20,2 (18,6–21,8)
	40–49	2.397 (22,4)	17,0 (15,1v19,1)	3.716 (21,9)	21,6 (19,7–23,5)	4.581 (22,0)	21,6 (19,9–23,3)
	50–59	1.871 (17,5)	16,0 (14,0–18,2)	2.924 (17,2)	19,6 (16,0–23,2)	3.676 (17,7)	18,6 (16,4–20,8)
	≥60	1.659 (15,5)	17,4 (14,9–20,2)	2.919 (17,2)	18,3 (14,7–21,8)	3.723 (17,9)	17,8 (15,2–20,4)
Sexo							
	Masculino	5.470 (47,0)	15,2 (13,9–16,6)	7.819 (46,1)	18,6 (16,9–20,3)	9.604 (46,1)	18,9 (17,4–205)
	Feminino	6.160 (53,0)	17,0 (15,7–18,4)	9.135 (53,9)	20,7 (19,0–22,4)	11.219 (53,9)	20,4 (18,8–-21,9)
Escolaridade							
	Superior	3.817 (32,8)	12,7 (11,3–14,2)	5.729 (33,8)	17,2 (16,3–18,1)	6.961 (33,4)	17,7 (16,8–18,6)
	EM completo	3.391 (29,2)	18,2 (16,4–20,1)	5.316 (31,4)	20,7 (19,6–21,7)	6.422 (30,8)	20,9 (19,9–21,9)
	EF completo/EM incompleto	1.578 (13,6)	19,3 (16,9–22,0)	2.107 (12,4)	22,1 (20,4–23,7)	2.667 (12,8)	21,8 (20,3–23,3)
	EF incompleto/sem instrução	2.844 (24,4)	17,0 (15,1–19,1)	3.802 (22,4)	20,9 (19,6–22,2)	4.773 (22,9)	19,8 (18,7–20,9)
Renda							
	≥R$ 2.500	2.563 (36,3)	12,3 (10,6–14,2)	3.682 (34,4)	15,7 (13,8–17,5)	4.431 (34,0)	16,5 (14,4–18,6)
	R$ 2.499 – R$ 1.430	1.828 (25,8)	15,4 (13,3–17,8)	2.727 (25,5)	18,9 (17,3–20,5)	3.253 (25,0)	19,3 (17,5–21,1)
	R$ 1.429- R$ 1.045	1.993 (28,2)	16,8 (14,8–19,1)	3.076 (28,7)	20,6 (18,9–22,2)	3.764 (28,9)	20,5 (18,5–22,6)
	R$ 1.044 – 0	687 (9,7)	19,2 (15,6–23,5)	1.224 (11,4)	19,9 (16,7–23,1)	1.580 (12,1)	19,5 (15,5–23,5)

* Prevalências e intervalos de confiança 95% para os testes RT-PCR ou teste rápido positivos para COVID-19.

IC: intervalo de confiança; EM: ensino médio; EF: ensino fundamental.

**Tabela 2:** Descrição da amostra conforme variáveis demográficas e socioeconômicas de acordo com a prevalência de teste de sangue positivo para COVID-19 em julho (n=6.886), setembro (n=10.668) e novembro (n=13.102), 2020.

Variáveis		Julho		Setembro		Novembro	
		n (%)	+COVID (IC95%)*	n (%)	+COVID (IC95%)*	n (%)	+COVID (IC95%)*
	Total	6.886 (100)	25,8 (24,2–27,5)	10.668 (100)	28,5 (27,3–29,7)	13.102 (100)	28,2 (27,4–28,9)
Raça							
	Branca	3.040 (44,2)	21,7 (19,6–23,9)	4.637 (43,5)	24,2 (22,3–26,1)	5.828 (44,5)	25,1 (23,4–26,8)
	Preta	530 (7,7)	23,6 (19,2–28,6)	876 (8,2)	29,3 (27,1–31,5)	1.068 (8,2)	28,3 (26,4–30,2)
	Parda	3.217 (46,7)	31,3 (28,8–33,9)	5.026 (47,1)	33,0 (21,6–34,4)	6.040 (46,1)	31,8 (20,5–33,1)
	Amarela	54 (0,8)	22,2 (9,5–43,6)	75 (0,7)	12,0 (3,1–20,9)	96 (0,7)	15,8 (6,9–24,7)
	Indígena	44 (0,6)	30,4 (14,2–53,6)	52 (0,5)	37,3 (22,3–52,3)	65 (0,5)	42,2 (27,2–57,2)
Faixa etária (anos)							
	18–29	1.138 (17,8)	24,1 (21,0–27,5)	1.971 (18,5)	27,6 (25,5–29,7)	2.387 (18,2)	28,1 (26,2–30,0)
	30–39	1.581 (24,8)	26,3 (23,2–29,6)	2.465 (23,1)	28,4 (26,4–30,4)	2.911 (22,2)	29,6 (27,8–31,4)
	40–49	1.441 (22,6)	26,7 (23,6–30,0)	2.263 (21,2)	29,2 (26,9–31,5)	2.845 (21,7)	28,3 (26,2–30,4)
	50–59	1.121 (17,5)	24,5 (21,3–28,0)	1.934 (18,1)	29,7 (26,9–32,5)	2.384 (18,2)	28,5 (26,7–30,3)
	≥60	1.103 (17,3)	27,0 (23,5–30,8)	2.035 (19,1)	28,9 (26,5–31,3)	2.575 (19,7)	27,4 (25,8–29,0)
Sexo							
	Masculino	3.185 (46,2)	24,8 (22,7–27,0)	4.892 (45,9)	28,2 (26,3–30,1)	5.954 (45,4)	27,6 (25,9–30,6)
	Feminino	3.701 (53,8)	26,7 (24,8–28,7)	5.776 (54,1)	29,2 (27,7–30,7)	7.148 (54,6)	29,1 (27,5–30,7)
Escolaridade							
	Superior	2.733 (39,7)	20,6 (18,6–22,9)	4.307 (40,4)	24,4 (22,6–26,2)	5.317 (40,6)	25,1 (23,6–26,6)
	EM completo	1.812 (26,3)	30,6 (27,7–33,7)	3.042 (28,5)	30,7 (28,4–33,0)	3.667 (28,0)	30,0 (28,1–31,9)
	EF completo/EM incompleto	815 (11,8)	28,1 (24,0–32,5)	1.177 (11,0)	33,4 (30,7–36,1)	1.491 (11,4)	31,4 (29,0–33,6)
	EF incompleto/sem instrução	1.526 (22,2)	29,6 (26,3–-33,1)	2.142 (20,1)	32,1 (29,5–34,7)	2.627 (20,1)	31,3 (29,5–33,3)
Renda							
	≥R$ 2,500	1.888 (43,9)	19,1 (16,7–21,8)	2.869 (41,3)	22,9 (20,8–25,0)	3.420 (40,5)	23,4 (21,5–25,3)
	R$ 2.499–R$ 1.430	964 (22,4)	25,6 (22,1–-29,5)	1.619 (23,3)	28,7 (25,5–31,9)	1.994 (23,6)	27,9 (24,9–30,9)
	R$ 1.429–R$ 1.045	1.088 (25,3)	25,0 (21,9–28,4)	1.772 (25,5)	28,8 (25,9–31,7)	2.162 (25,6)	28,6 (26,4–30,8)
	R$ 1.044–0	364 (8,4)	31,1 (25,0–37,8)	684 (9,9)	33,0 (27,3–38,7)	872 (10,3)	29,7 (25,5–33,9)

* Prevalências e intervalos de confiança 95% para os testes RT-PCR ou teste rápido positivos para COVID-19.

IC: intervalo de confiança; EM: ensino médio; EF: ensino fundamental.

A Tabela 3 apresenta as RP e o IC95% da análise bruta e ajustada entre a realização dos três diferentes testes (RT-PCR, teste rápido e testagem sanguínea) durante os meses de julho, setembro e novembro (2020) e as variáveis de exposição. Após realizados os ajustes para os potenciais fatores de confusão, foi observado um efeito significativo da raça/etnia na testagem de COVID-19 através do teste RT-PCR entre os participantes indígenas em novembro (RP: 1,90; IC95% 1,07–3,38), visto que esse grupo apresentou maior probabilidade de infecção por SARS-CoV-2 via teste RT-PCR comparado aos indivíduos brancos. Participantes de etnia parda apresentaram maior RP de testagem positiva através do RT-PCR em setembro (RP: 1,20; IC95% 1,10–1,30), comparado aos sujeitos brancos. Ainda, indivíduos com menor renda (R$ 0–1) apresentaram maior probabilidade de apresentar teste RT_PCR para verificar a ocorrência de infecção por COVID-19 do que aqueles com renda familiar mais elevada (≥R$ 2.500) em setembro (RP: 1,87; IC95%1,15–2,67) e novembro (RP: 1,69; IC95% 1,16–3,06).

**Tabela 3:** Análise bruta e ajustada para os testes RT-PCR, teste rápido e de sangue positivo para COVID-19 em julho, setembro e novembro, 2020.

Variáveis		Julho		Setembro		Novembro	
		RP_BRUTA_ (IC95%)	RP_AJUSTADA_ (IC95%)	RP_BRUTA_ (IC95%)	RP_AJUSTADA_ (IC95%)	RP_BRUTA_ (IC95%)	RP_AJUSTADA_ (IC95%)
Teste RT-PCR							
Raça							
	Branca	1,00	1,00	1,00	1,00	1,00	1,00
	Preta	1,05 (0,86–1,27)	1,09 (0,88–1,35)	1,06 (0,92–1,22)	1,05 (0,91–1,21)	0,96 (0,85–1,09)	0,97 (0,86–1,10)
	Parda	1,11 (0,98–1,25)	1,05 (0,92–1,21)	1,21 (1,11–1,31)	1,20 (1,10–1,30)	1,18 (1,11–1,27)	1,20 (1,12–1,29)
	Amarela	0,85 (0,34–2,16)	0,72 (0,26–1,99)	0,46 (0,26–0,81)	0,44 (0,25–0,77)	0,65 (0,41–1,02)	0,65 (0,41–1,02)
	Indígena	1,35 (0,65–2,79)	1,39 (0,37–5,14)	1,73 (1,09–3,33)	1,63 (0,85–3,15)	1,92 (1,08–3,40)	1,90 (1,07–3,38)
Renda							
	≥R$ 2.500	1,00	1,00	1,00	1,00*	1,00	1,00
	R$ 2.499–R$ 1.430	0,98 (0,84–1,15)	0,90 (0,76–1,05)	1,09 (0,97– 1,23)	1,05 (0,93–1,19)	0,98 (0,89–1,08)	0,97 (0,87–1,08)
	R$ 1.429–R$ 1.045	0,98 (0,83–1,16)	0,90 (0,75–1,07)	1,14 (1,01–1,28)	1,08 (0,94–1,24)	1,05 (0,95–1,15)	1,04 (0,93–1,16)
	R$ 1.044–0	0,90 (0,66–1,22)	0,78 (0,56–1,08)	0,99 (0,82–1,19)	1,87 (1,15–2,67)	1,31 (0,98–1,76)	1,69 (1,16–3,06)
Teste rápido							
Raça							
	Branca	1,00	1,00/	1,00*	1,00	1,00	1,00*
	Preta	1,24 (0,98–1,57)	1,09 (0,84–1,42)	1,31 (1,15–1,51)	1,31 (1,02–1,32)	1,17 (1,04–1,33)	1,16 (1,03–1,32)
	Parda	1,44 (1,26–1,64)	1,22 (1,04–1,44)	1,47 (1,35–1,59)	1,47 (1,35–1,60)	1,42 (0,91–2,26)	1,41 (0,90–2,21)
	Amarela	0,97 (0,45–2,07)	1,04 (0,43–2,51)	1,17 (0,67–2,02)	1,07 (0,58–1,87)	1,16 (0,73–1,85)	1,15 (0,72–1,84)
	Indígena	2,66 (1,69–4,19)	2,45 (1,48–4,08)	2,95 (1,96–4,45)	2,53 (1,74–4,41)	1,39 (1,29–1,50)	1,23 (1,11–1,86)
Renda							
	≥R$ 2.500	1,00	1,00	1,00*	1,00*	1,00	1,00*
	R$ 2.499–R$ 1.430	1,25 (1,02–1,53)	1,14 (0,93–1,41)	1,26 (1,11–1,44)	1,27 (1,11–1,45)	1,19 (1,07–1,36)	1,22 (1,08–1,37)
	R$ 1.429–R$ 1.045	1,36 (1,13–1,65)	1,16 (0,94–1,43)	1,39 (1,22–1,58)	1,42 (1,25–1,61)	1,31 (1,17–1,47)	1,33 (1,19–1,49)
	R$ 1.044–0	1,56 (1,22–2,00)	1,20 (1,00–1,57)	1,35 (1,13–1,59)	1,38 (1,17–1,64)	1,22 (1,06–1,42)	1,25 (1,08–1,45)
Teste sangue							
Raça							
	Branca	1,00	1,00	1,00	1,00	1,00	1,00*
	Preta	1,09 (0,87–1,36)	1,07 (0,82–1,38)	1,30 (1,11–1,53)	1,30 (1,11–1,53)	1,18 (1,02–1,37)	1,18 (1,02–1,37)
	Parda	1,44 (1,28–1,63)	1,30 (1,11–1,53)	1,84 (1,41–1,69)	1,54 (1,41–1,69)	1,39 (1,28–1,51)	1,39 (1,28–1,51)
	Amarela	1,02 (0,48–2,18)	0,78 (0,31–1,99)	0,43 (0,21–0,86)	0,43 (0,21–0,86)	0,56 (0,32–0,98)	0,56 (0,32–0,98)
	Indígena	1,40 (0,74–2,68)	1,14 (0,53–2,48)	1,36 (1,02–3,29)	1,86 (1,05–3,29)	2,18 (1,32–3,59)	2,11 (1,12–3,59)
Renda							
	≥R$ 2.500	1,00	1,00	1,00	1,00*	1,00	1,00*
	R$ 2.499–R$ 1.430	1,34 (1,10–1,64)	1,28 (1,05–1,56)	1,36 (1,18–1,56)	1,38 (1,20–1,59)	1,27 (1,12–1,44)	1,28 (1,13–1,45)
	R$ 1.429–R$ 1.045	1,31 (1,09–1,57)	1,21 (1,00–1,46)	1,37 (1,19–1,57)	1,41 (1,22–1,62)	1,31 (1,16–1,48)	1,34 (1,18–1,52)
	R$ 1.044–0	1,63 (1,27–2,08)	1,39 (1,08–1,79)	1,67 (1,39–2,00)	1,72 (1,43–2,07)	1,38 (1,17–1,63)	1,43 (1,21–1,69)

* Ajustado para escolaridade, sexo e faixa etária.

Os participantes autodeclarados indígenas apresentaram maior chance de serem diagnosticados com COVID-19 por meio de testes rápidos do que participantes de raça branca em todos os meses de acompanhamento: julho (RP: 2,45; IC95% 1,48–4,08), setembro (RP: 2,53; IC95% 1,74–4,41), novembro (RP: 1,23; IC95% 1,11–1,86). A renda média mensal dos participantes esteve associada com a prevalência de testagem rápida para COVID-19: os participantes com menor renda mensal apresentaram maior probabilidade de realizar teste rápido do que aqueles que ganham ≥2,500 reais mensais em julho, setembro e novembro (2020). Foi identificado que os participantes pardos tinham maiores prevalências de testagem positiva para COVID-19 através de exames sanguíneos em todos os meses de acompanhamento: julho (RP: 1,30; IC95% 1,11–1,53), setembro (RP: 1,54; IC95% 1,41–1,69), novembro (RP: 1,39; IC95% 1,28–1,51), comparado aos sujeitos brancos. Indivíduos autodeclarados como indígenas também apresentaram maior RP de testagem positiva para COVID-19 por testes sanguíneos, mas apenas em setembro (RP: 1,86; IC95% 1,05–3,29) e novembro (RP: 2,11; IC95% 1,12–3,59) de 2020. A menor renda familiar mensal esteve associada à menor probabilidade de resultado positivo no teste sanguíneo em todos os meses de acompanhamento do que aqueles com renda ≥R$ 2.500/mês (Tabela 3).

## DISCUSSÃO

Este estudo retrospectivo utilizou os dados do Pnad COVID-19 para investigar as desigualdades relacionadas a raça e renda no histórico de testagem para COVID-19 de acordo com as modalidades diagnósticas em adultos durante a primeira onda da pandemia no Brasil. Os resultados indicam que indivíduos indígenas e com menor renda média mensal apresentaram maior probabilidade de serem diagnosticados com COVID-19, independente do teste utilizado e o período de realização do exame.

Neste estudo, indivíduos não brancos, especialmente aqueles pardos e(ou) indígenas, apresentaram maior soroprevalência para COVID-19, quando comparado com os sujeitos brancos. Esse achado pode ser justificado pelas condições sociais que as minorias populacionais estão expostas, predispondo a maior taxa de infecção pela doença nesse grupo^18^. Tal resultado corrobora a situação histórica de vulnerabilidade vivida pela população indígena, em particular, ao qual apresentou maiores quadros infecciosos no passado, como na gripe espanhola, infecção pelo vírus da H1N1 e SARS-CoV^19^.

De fato, esse grupo populacional é fortemente impactado pelas iniquidades nos determinantes sociais da saúde. Além das barreiras culturais e geográficas, os indígenas apresentam quadros mais elevados de pobreza, desnutrição, baixa escolaridade, dificuldade de acesso aos serviços de saúde e precarização dos sistemas de saneamento básico^19,20^. Além disso, é importante destacar a característica de isolamento geográfico comum nessa população como potencial obstáculo ao acesso às medidas de atenção em saúde voltadas para o combate à pandemia. Ademais, fatores imunológicos característicos dos indígenas e a presença das doenças crônicas aumentam a suscetibilidade a surtos de doenças infecciosas, tornando os povos mais vulneráveis^21^. Agrava-se essa realidade quando a exposição dessa comunidade é associada a omissão governamental de anteparo diante da doença, como reportado no relatório elaborado pelo Conselho Nacional de Saúde (CNS) e pelo Conselho Nacional de Direitos Humanos (CNDH)^22^.

Os resultados deste estudo evidenciam uma associação significativa entre a testagem via RT-PCR e exame sanguíneo e a renda mensal dos participantes. Indivíduos com menor renda apresentaram maior chance de testagem positiva através dessas modalidades diagnósticas. No Brasil, a disseminação do vírus da COVID-19 teve início em indivíduos de classe econômica mais elevada e posteriormente o vírus se espalhou rapidamente entre as pessoas de classe econômica menos favorecida, visto que parte desses indivíduos realizaram a manutenção das suas atividades diárias por necessidade de subsistência^23,24^. Estudos têm reportado que as condições socioeconômicas desfavoráveis, reduzido nível de escolaridade e o maior número de residentes no domicílio podem predispor a maior taxa de infecção pela COVID-19^12^. A Comissão Econômica para a América Latina e o Caribe (Cepal) sugere que o empobrecimento da população brasileira nos últimos anos pode ter aumentado o impacto da COVID-19 no país^25^. Grupos desfavorecidos tendem a ter ocupações menos estruturadas e com uma renda insuficiente para sua sobrevivência^26–28^., geralmente inseridos em ocupações que não oferecem a possibilidade de trabalhar em casa, necessitando utilizar o transporte público para a locomoção e, assim, aumentando o contato entre as pessoas^29,30^. Ademais, a insuficiente testagem realizada no Brasil durante o período da Pnad COVID-19, resultado do escasso número de testes disponíveis no SUS e omissão do governo brasileiro na aquisição, resultou na compra dos testes de forma individual, afastando as populações desfavorecidas do diagnóstico da doença^26,28,29^. Todavia, nos desfechos dos testes realizados, a maior positividade é destacada na população mais sensível economicamente.

Os resultados apresentados neste estudo devem ser interpretados à luz de suas limitações. A principal limitação desta investigação se refere à natureza transversal do estudo, que não permite a inferência da causalidade nas associações identificadas. O uso de dados autorreportados também pode ser considerado uma limitação, uma vez que a percepção subjetiva é fruto do comportamento verbal, e esse, por sua vez, é reforçado pelo ambiente ao qual o indivíduo está inserido^25^.

Do mesmo modo, cabe destacar as limitações dadas pelos resultados dos testes. O teste RT-PCR está indicado para pacientes sintomáticos na fase aguda da doença entre o terceiro e o sétimo dia, assim devem-se considerar possíveis resultados falsos negativos que ocorrem quando a quantidade de genoma viral coletado é insuficiente ou o período da janela de tempo da replicação viral é perdido^5^. Entretanto esse teste possui alta sensibilidade e especificidade, respectivamente 97,2 e 98,9%^5^.

Além disso, é possível realizar o diagnóstico de COVID-19 com base na resposta imune à infecção pelo SARS-CoV 2, através dos testes sorológicos imunocromatográficos para detecção rápida dos anticorpos IgG/IgM, em amostras de sangue, soro ou plasma dos indivíduos^5^. A principal limitação dessa ferramenta é a necessidade da realização, a partir do oitavo dia do início dos sintomas. Assim, o resultado da doença que se apoia nessa detecção possivelmente ocorrerá em um momento de recuperação do paciente, aumentando o desafio dos serviços de vigilância e controle da transmissão. Os testes sorológicos IgM e IgG possuem sensibilidade de 84,5% e especificidade de 91,6%^4,5^., porém os testes rápidos imunocromatográficos apresentavam baixa sensibilidade, particularmente no início da pandemia^31^.

Importa destacar que a sensibilidade de um teste diagnóstico diz respeito à capacidade de identificar corretamente o resultado positivo da doença, e a especificidade aponta a capacidade de identificar a ausência^4,5^.

Com base numa revisão de literatura realizada pelos autores, até o momento, não foram publicados estudos que procuraram validar essas questões autorreferidas de resultados positivos para COVID-19. Estudos com delineamentos de coorte e testes bioquímicos poderiam melhor ilustrar as desigualdades no contágio pela doença. Ademais, é importante considerar que, em abril de 2020, as terras indígenas mais vulneráveis eram aquelas localizadas às margens dos grandes centros urbanos, como Manaus, eixo Rio Branco-Porto Velho, Fortaleza, Salvador e capitais do Sul e Sudeste do Brasil. Assim, considerando a atuação da Pnad COVID-19 nos grandes centros urbanos, sugere-se que essas descobertas podem representar um quadro menor da realidade, uma vez que não alcança efetivamente aldeias e terras indígenas em regiões isoladas do país. Por fim, os resultados aqui descritos são válidos e robustos, somando a um campo pouco explorado na literatura brasileira.

Este estudo identificou associação significativa entre a etnia/raça e a situação econômica com resultado positivo para COVID-19 em adultos brasileiros. Esse panorama reflete uma situação de vulnerabilidade nesses grupos e demonstra a necessidade da elaboração e expansão de políticas públicas mais equitativas que contemplem grupos mais vulneráveis em contexto de crise de saúde pública.
